# Associations Between Lipoprotein Subfractions and Area and Density of Abdominal Muscle and Intermuscular Adipose Tissue: The Multi-Ethnic Study of Atherosclerosis

**DOI:** 10.3389/fphys.2021.713048

**Published:** 2021-09-27

**Authors:** Megan M. Marron, Matthew Allison, Alka M. Kanaya, Britta Larsen, Alexis C. Wood, David Herrington, Philip Greenland, Iva Miljkovic

**Affiliations:** ^1^Department of Epidemiology, Graduate School of Public Health, University of Pittsburgh, Pittsburgh, PA, United States; ^2^Department of Family Medicine and Public Health, School of Medicine, University of California, San Diego, San Diego, CA, United States; ^3^Department of Medicine, University of California, San Francisco, San Francisco, CA, United States; ^4^United States Department of Agriculture/Agriculture Research Service Children’s Nutrition Research Center, Baylor College of Medicine, Houston, TX, United States; ^5^Department of Internal Medicine, Wake Forest School of Medicine, Winston-Salem, NC, United States; ^6^Department of Preventive Medicine, Feinberg School of Medicine, Northwestern University, Chicago, IL, United States

**Keywords:** skeletal muscle, myosteatosis, lipids, metabolism, lipidomics, lipoproteins

## Abstract

Skeletal muscle quantity and quality decrease with older age, which is partly attributed to ectopic fat infiltration and has negative metabolic consequences. To inform efforts to preserve skeletal muscle with aging, a better understanding of biologic correlates of quantity and quality of muscle and intermuscular adipose tissue (IMAT) is needed. We used targeted lipidomics of lipoprotein subfractions among 947 Multi-Ethnic Study of Atherosclerosis participants to provide a detailed metabolic characterization of area and density of abdominal muscle and IMAT. Serum lipoprotein subfractions were measured at the first visit using 1H-Nuclear Magnetic Resonance spectroscopy. Muscle and IMAT area (cm^2^) and density (Hounsfield units) were estimated at visit 2 or 3 using computed tomography of the total abdominal, locomotion (psoas), and stabilization (paraspinal, oblique, rectus abdominis) muscles. We identified lipoprotein subfractions associated with body composition using linear regression adjusting for demographics, lifestyle, and multiple comparisons. Among 105 lipoprotein subfractions, 24 were associated with total muscle area (absolute standardized regression coefficient range: 0.07–0.10, *p*-values ≤ 0.002), whereas none were associated with total muscle density. When examining muscle subgroups, 25 lipoprotein subfractions were associated with stabilization muscle area, with associations strongest among the obliques. For total IMAT area, there were 27 significant associations with lipoprotein subfractions (absolute standardized regression coefficient range: 0.09–0.13, *p*-values ≤ 0.002). Specifically, 27 lipoprotein subfractions were associated with stabilization IMAT area, with associations strongest among the oblique and rectus abdominis muscles. For total IMAT density, there were 39 significant associations with lipoprotein subfractions (absolute standardized regression coefficient range: 0.10–0.19, *p*-values ≤ 0.003). Specifically, 28 and 33 lipoprotein subfractions were associated with IMAT density of locomotion and stabilization (statistically driven by obliques) muscles, respectively. Higher VLDL (cholesterol, unesterified cholesterol, phospholipids, triglycerides, and apolipoprotein B) and lower HDL (cholesterol and unesterified cholesterol) were associated with higher muscle area, higher IMAT area, and lower IMAT density. Several associations between lipoprotein subfractions and abdominal muscle area and IMAT area and density were strongest among the stabilization muscles, particularly the obliques, illustrating the importance of examining muscle groups separately. Future work is needed to determine whether the observed associations indicate a lipoprotein profile contributing to worse skeletal muscle with fat infiltration.

## Introduction

Skeletal muscle and adipose tissue are endocrine organs (Ahima and Park, 2015; Choe et al., 2016) involved in multiple physiologic and pathologic processes (Miljkovic and Zmuda, 2010). Specifically, skeletal muscle is central to glucose and insulin homeostasis, fatty acid oxidation, and amino acid storage (Ferrannini et al., 1985; Kelley et al., 1993; Richard et al., 2000; Miljkovic and Zmuda, 2010), while adipose tissue is not only critical for energy storage, but also for maintaining insulin sensitivity and systemic homeostasis (Nakamura et al., 2014) through secretion of hormones such as leptin, adiponectin, and resistin (Richard et al., 2000). However, with aging and excess caloric consumption, excess fat can accumulate in ectopic locations, including in and around skeletal muscle. This accumulation disrupts normal secretion and sensing of adipocyte-derived hormones and adipokines, triggering systemic low-grade inflammation (Richard et al., 2000). Ectopic fat accumulation also impairs normal skeletal muscle metabolic function (Miljkovic and Zmuda, 2010; Correa-de-Araujo et al., 2020) and is partly responsible for adverse decreases in area (quantity) and density (quality) of skeletal muscle with aging (Evans, 1995; Goodpaster et al., 2006).

Ectopic adipose tissue can accumulate fat differentially based on fiber content of muscle. For example, type I fibers (slow-twitch fibers) have a greater fat content than type II fibers (fast-twitch fibers) (He et al., 2001). Abdominal skeletal muscles can be broadly classified as muscles of stabilization vs. muscle of locomotion, where the stabilization muscles consist more of type I fibers (Häggmark and Thorstensson, 1979) and the locomotion muscles consist more of type II fibers (Arbanas et al., 2009). To inform efforts to slow aging-related muscle atrophy and prevent muscle fat infiltration, a better understanding of biologic correlates of quantity and quality of skeletal muscle, as well as intermuscular adipose tissue (IMAT), and how the identified biologic correlates differ based on different subgroups of skeletal muscle is needed.

Lipidomics is the large-scale study of lipids, which are chemically diverse and have increasingly been shown to have biologic relevance to multiple physiologic processes (Rampler et al., 2018). Blood-based lipidomics has the potential to provide a better understanding of the biologic correlates of skeletal muscle and IMAT since it directly measures the circulating lipids left behind from cellular processes (Clish, 2015; The European Bioinformatics Institute, 2015). As such, we utilized available data on targeted lipidomics of concentrations of total cholesterol, unesterified cholesterol (free cholesterol), phospholipids, triglycerides, apolipoprotein (apo) A-I, apo A-II, and apo B for several lipoprotein classes and subclasses. In addition, we utilized measures of area and density of skeletal muscle and IMAT derived from computed tomography scans of the total abdominal, locomotion (psoas), and stabilization (paraspinal, oblique, rectus abdominis) muscles among participants from the Multi-Ethnic Study of Atherosclerosis (MESA). Our specific aims were to (1) identify lipoprotein subfractions associated with quantity and quality of abdominal muscle and IMAT and (2) determine if the identified associations were stronger in a specific abdominal muscle subgroup. We hypothesized lower density lipoprotein subfractions would be positively associated with quantity, but inversely associated with quality of muscle and IMAT, and higher density lipoprotein subfractions would be inversely associated with quantity, but positively associated with quality of muscle and IMAT.

## Materials and Methods

### Multi-Ethnic Study of Atherosclerosis

The MESA is a prospective cohort of 6,814 adults ages 44–84 years who were free of clinically apparent cardiovascular disease at recruitment and self-reported as White (38%), Black (28%), Asian American (12%), or Hispanic (22%). MESA was originally designed to investigate the prevalence, correlates, and progression of subclinical cardiovascular disease among a diverse population-based cohort (Bild et al., 2002). Participants were recruited from six U.S. communities during July 2000 to August 2002 for an initial visit and returned for follow-up exams after approximately 2, 4, 5, 10, and 16 years. The MESA and its ancillary studies were approved by the Institutional Review Board of each participating site. All participants provided written informed consent.

An ancillary study quantified lipoprotein subfractions utilizing fasting serum samples collected at the first visit (2000–2002) among 3809 randomly selected MESA participants. A second ancillary study assessed body composition for 1970 randomly selected MESA participants at the second or third visit (2002–2005). There were 1031 participants with complete information on lipoprotein subfractions *and* area and density of abdominal muscle and IMAT. Among these 1031 participants, 84 were missing additional covariates of interest. Thus, our analytic sample included 947 participants. Among the 947 participants, body composition was assessed 2.6 years, on average, after the first visit (median = 3.0 years, range: 1.1–4.8 years).

### Lipoprotein Subfractions

In fasting serum that was collected at the first visit, we measured concentrations of total and unesterified cholesterol, phospholipids, triglycerides, apo A-I, apo A-II, and apo B for several lipoprotein main classes and subclasses (a total of 150 measures). Samples were prepared using the Bruker standard method (Dona et al., 2014). Lipoprotein subfractions were quantified using Bruker lipoprotein subclass analysis (Bruker Biospin, Germany) with adapted methods (Petersen et al., 2005) and 1H-Nuclear Magnetic Resonance spectroscopy. Lipoprotein subfractions were separated by serial ultracentrifugation (Petersen et al., 2005) and identified from methyl peak deconvolution near 0.89 ppm (Neeland et al., 2019). The following lipoprotein classes were measured: very low-density lipoproteins (VLDL; < 1.006 kg/L), intermediate-density lipoproteins (IDL; 1.006–1.019 kg/L), low-density lipoproteins (LDL; 1.019–1.063 kg/L), and high-density lipoproteins (HDL; 1.063–1.210 kg/L) (Petersen et al., 2005). In addition, subclasses of VLDL, LDL, and HDL were also measured. VLDL and LDL were categorized into six subclasses and HDL was categorized into four subclasses. All subclasses were numbered according to increasing density (Lindgren et al., 1972; Neeland et al., 2019).

### Body Composition

Abdominal computed-tomography scans were performed at the second or third visit. For this report, we used pixel intensities from a single scan at the L4/L5 vertebral junction. Slices were processed using MIPAV 4.1.2 (National Institutes of Health, Bethesda, MD), which estimated area (cm^2^) and density (Hounsfield units, Hu) of total abdominal muscle and IMAT using a semi-automated method (Vella and Allison, 2018; Vella et al., 2020). Total abdominal measures were the sum of the four muscle groups: right and left psoas, paraspinal, oblique, and rectus abdominis. Abdominal muscle groups were examined individually, as well as categorized as muscles of locomotion (psoas) or stabilization (paraspinal, oblique, and rectus abdominus). The paraspinal muscle group included mostly the erector spinae, but also quadratus lumborum, spinalis, longissimus, iliocostalis, and multifidus. The oblique muscle group included the internal oblique, external oblique, and transverse abdominus.

Adipose tissue was identified as tissue between −190 and −30 HU, while muscle tissue was identified as tissue between 0 and 100 HU. After the tissue type was identified, muscle area was recorded as the number of pixels between 0 and 100 HU within the respective area of interest and IMAT area was the number of pixels between −190 and −30 HU. Density was recorded as the average HU of the pixels within the respective area of interest. Lower density muscle tissue indicated worse quality muscle with greater fat infiltration. Similarly, lower density IMAT indicated worse quality fat within the muscle fascia, with a higher lipid content per adipocyte and lower vascularity (Rosenquist et al., 2015). High inter- and intra-rater reliability of 0.99 was obtained for total abdominal area and high inter- and intra-rater reliability ranging from 0.93 to 0.98 were obtained for all abdominal muscle groups (Vella et al., 2020).

### Participant Characteristics

Participants self-reported age, gender, and race/ethnicity as either White, Black, Asian American, or Hispanic. Participants brought in all medications used in the past 2 weeks for an inventory. Body mass index was calculated using height recorded to the nearest 0.1 cm and weight recorded to the nearest 0.5 kg. Waist circumference was measured using a Gulick II anthropometric tape at the umbilicus and hip circumference at the maximum circumference of the buttocks. Physical activity was assessed using the MESA Typical Week Physical Activity Survey. Moderate-to-vigorous activity was the sum of the metabolic equivalent (MET)-minutes/week doing the following activities that required moderate-to-heavy effort: household chores, lawn/yard/garden/farm work, caregiving, transportation, walking, dancing, team or dual sport activities, conditioning activities, and occupational and volunteer activities. Sedentary behavior was the sum of the MET-minutes/week watching television, reading, knitting, sewing, doing nothing, non-work recreational computer usage, and light effort/sitting at work. Dietary fat was assessed from self-report of usual diet over the past year using a 120-item Food Frequency Questionnaire that was developed in the Block format (Block et al., 1990) and modified from the validated Food Frequency Questionnaire used in the Insulin Resistance Atherosclerosis Study (Mayer-Davis et al., 1999). The alternate healthy eating index (McCullough et al., 2002) was calculated using information on servings per day of vegetables, fruit, nuts and soy protein, ratio of white to red meat, cereal fiber, trans fat, ratio of polyunsaturated to saturated fats, multivitamin use, and alcohol intake.

Participants were instructed to fast for 12 h prior to their study visit, where phlebotomy was performed. Serum glucose was measured using the Vitros analyzer (Johnson & Johnson Clinical Diagnostics, Inc., Rochester, NY). Diabetes was defined as fasting glucose ≥ 126 mg/dL or taking medication for diabetes. Plasma triglycerides and total and HDL cholesterol were measured using a standardized kit (Roche Diagnostics, Rotkreuz, Switzerland). LDL cholesterol was estimated using the Friedewald equation (Friedewald et al., 1972). Serum insulin was measured using the Linco Human Insulin Specific RIA kit (Linco Research, St. Charles, MO). Serum interleukin-6 was measured using ultrasensitive ELISA from R&D Systems (Minneapolis, MN). Serum C-reactive protein was measured using the BNII nephelometer (Deerfield, IL). Serum creatinine was measured using colorimetry and Vitros 950 analyzer (Johnson & Johnson Clinical Diagnostics Inc., Rochester, NY).

### Statistical Analysis

Study characteristics were described using mean and standard deviations for continuous measures and frequency and percentages for categorical measures. Non-normally distributed lipoprotein subfractions were log transformed. All lipoprotein subfractions were standardized to a mean of zero and standard deviation of one. We identified lipoprotein subfractions that were significantly associated with the different measures of abdominal body composition using linear regression adjusting for age, gender, race/ethnicity, alternate healthy eating index, moderate-to-vigorous physical activity, sedentary behavior, and lipid-lowering medication use. We accounted for multiple comparisons using a Benjamini-Hochberg correction with a 1% false discovery rate. We did not further adjust for additional body composition measures (e.g., visceral adipose tissue) since lipoprotein subfractions were assumed to be common causes of the additional body composition measures (e.g., visceral adipose tissue) and of the body composition outcomes examined in this report. That is, these additional body composition measures could either be simply common descendants of lipoprotein subfractions (i.e., not directly impacting the body composition outcomes examined in this report) or they could be mediators of the relationships between lipoprotein subfractions and the body composition outcomes examined in this report, where further adjusting for a mediator would cause overadjustment bias (Schisterman et al., 2009). In addition, we did not further adjust for body mass index nor waist circumference since these body size measures are common descendants of the body composition outcomes examined in this report. Adjusting for an outcome’s descendant can also lead to a biased estimate of the true effect (Schisterman et al., 2009). However, we did examine whether associations between lipoprotein subfractions and abdominal body composition differed after adjusting for height, i.e., a measure of body size that is *not* a common descendant of body composition, but rather a potential confounder of the relationship between lipoprotein subfractions and body composition.

We used volcano plots to visualize the pattern and the amount and type of lipoprotein subfractions that were significantly associated with abdominal body composition. Specifically, we plotted the adjusted standardized regression estimate for the association between a single lipoprotein subfraction and a single abdominal body composition measure (x-axis) against the significance of that association (y-axis). Information used in the volcano plots were adjusted for age, gender, race/ethnicity, alternate healthy eating index, moderate-to-vigorous physical activity, sedentary behavior, and lipid-lowering medication use. A reference line was used to indicate a threshold for *p*-values less than 0.05. A second reference line was included to indicate false discovery rates less than 1%, when this level of significance was reached. We also examined plots of the standardized regression estimate (y-axis) and 95% confidence intervals for the association between a lipoprotein subfraction and abdominal body composition by lipoprotein subfraction content (x-axis) and organized from lowest to highest lipoprotein subfraction density. This helped visualize the pattern in associations between lipoprotein subfractions and abdominal body composition as the lipoprotein subfractions became denser.

## Results

**FIGURE 1:**
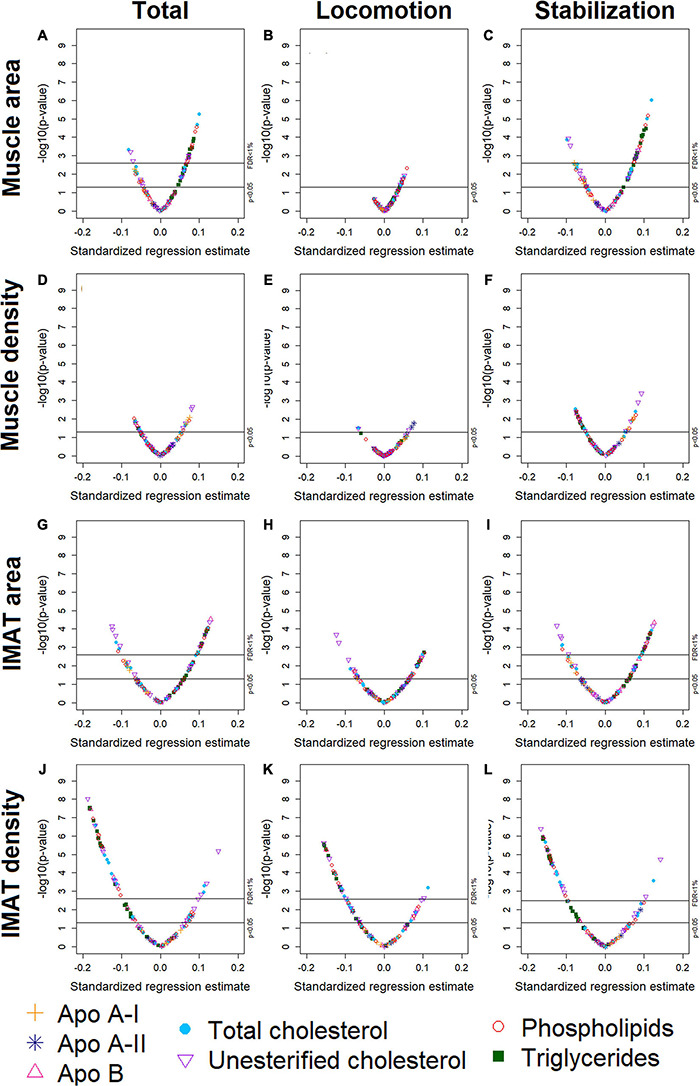
Volcano plots of associations between 105 lipoprotein subfractions and total muscle area **(A)**, locomotion muscle area **(B)**, stabilization muscle area **(C)**, total muscle density **(D)**, locomotion muscle density **(E)**, stabilization muscle density **(F)**, total IMAT area **(G)**, locomotion IMAT area **(H)**, stabilization IMAT area **(I)**, total IMAT density **(J)**, locomotion IMAT density **(K)**, and stabilization IMAT density **(L)** among 947 MESA participants.

Participants (*n* = 947) were 44–84 years old (mean age: 63, standard deviation: 9.8), 51% men, 40% White, 16% Black, 16% Chinese American, and 27% Hispanic American (Table 1). With adjustment for age, gender, race/ethnicity, alternate healthy eating index, moderate/vigorous physical activity, sedentary behavior, lipid-lowering medication use, and multiple comparisons, there were 24 lipoprotein subfractions significantly associated with total muscle area, 27 lipoprotein subfractions significantly associated with total IMAT area, and 39 lipoprotein subfractions significantly associated with total IMAT density (Figures 1A,G,J, respectively). There were no significant associations between lipoprotein subfractions and total muscle density after accounting for multiple comparisons (Figure 1D). When examining density of muscle subgroups, there were also no significant associations between lipoprotein subfractions and locomotion muscle density (Figure 1E) nor stabilization muscle density (Figure 1F). Higher VLDL (total cholesterol, unesterified cholesterol, phospholipids, triglycerides, and apo B) *and* lower HDL (total and unesterified cholesterol) were associated with higher total muscle area, higher total IMAT area, and lower total IMAT density (Table 2). Adjusted associations between lipoprotein subfractions and abdominal body composition did not differ significantly by gender and/or race after accounting for multiple comparisons nor did associations differ after accounting for body size by adjusting for height.

**TABLE 1:** Study characteristics at visit 1 among 947 MESA participants.

**Mean (standard deviation) or frequency (percent)**	**Overall (*N* = 947)**
Age (years)	63 (9.8)
Men	480 (51%)
Race/ethnicity:	
Black	153 (16%)
Asian American	151 (16%)
Hispanic	260 (27%)
White	383 (40%)
More than high school education	396 (42%)
Current smoker	111 (12%)
Moderate/vigorous activity (MET-minutes/week)	5,679 (5,585) Median = 4,050
Sedentary behavior (MET-minutes/week)	2,478 (1,580) Median = 2,160
Number of medications	3.2 (2.9) Median = 3
Lipid-lowering medication use	169 (18%)
Diabetes	104 (11%)
Alternate health eating index	44 (11)
Percent total calories from fat	31 (7.2)
Blood-based biomarkers:	
Fasting insulin (mU/L)	10.3 (6.9) Median = 8.2
Fasting glucose (mg/dL)	97.8 (29) Median = 90
Total cholesterol (mg/dL)	197 (35)
HDL cholesterol (mg/dL)	51 (15)
LDL cholesterol (mg/dL)	119 (31)
Triglycerides (mg/dL)	140 (87) Median = 119
Creatinine (mg/dL)	0.98 (0.2) Median = 0.92
Interleukin-6 (pg/mL)	1.4 (1.1) Median = 1.1
C-reactive protein (mg/L)	3.4 (4.8) Median = 1.8
Weight (lbs)	170 (35)
Height (cm)	166 (10)
Body mass index (kg/m^2^)	28 (4.9)
Waist circumference (cm)	97 (13)
Hip circumference (cm)	104 (10)
Abdominal body composition:	
*Total muscle area (cm* ^2^ *):*	97 (28)
Psoas muscle area	24 (7.4)
Paraspinal muscle area	33 (11)
Oblique muscle area	31 (10)
Rectus abdominus muscle area	9 (4.3)
*Total IMAT area (cm* ^2^ *):*	25 (12)
Psoas IMAT area	2 (1.6)
Paraspinal IMAT area	14 (6.8)
Oblique IMAT area	6.2 (4.7)
Rectus abdominus IMAT area	2.9 (1.9)
*Total muscle density (Hu):*	337 (44)
Psoas muscle density	101 (10)
Paraspinal muscle density	81 (13)
Oblique muscle density	81 (12)
Rectus abdominus muscle density	75 (17)
*Total IMAT density (Hu):*	−484 (35)
Psoas IMAT density	−115 (13)
Paraspinal IMAT density	−133 (13)
Oblique IMAT density	−119 (12)
Rectus abdominus IMAT density	−118 (11)

*HDL, high-density lipoprotein; LDL, low-density lipoprotein; IMAT, intermuscular adipose tissue; MET, metabolic equivalent; mU/L, milliunits per liter; mg/dL, milligrams per deciliter; pg/mL, picograms per milliliter; mg/L, milligrams per liter; lbs, pounds; cm, centimeter; kg/m^2^, kilograms per meters squared; Hu, Hounsfield units.*

**TABLE 2:** Adjusted standardized regression estimates of the associations between lipoprotein subfractions and total abdominal body composition for the subset of 46 lipoprotein subfractions that were significantly associated with at least one of the total abdominal body composition measures.

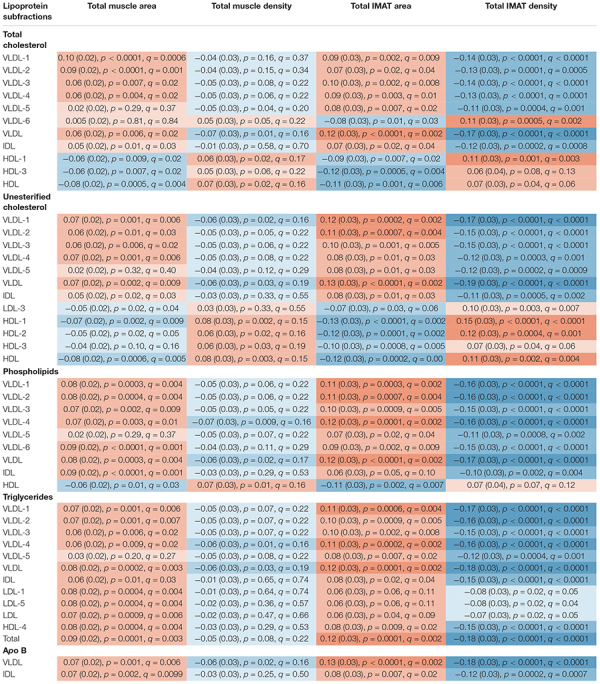

*Associations were adjusted for age, gender, race, alternate healthy eating index, moderate-to-vigorous physical activity, sedentary behavior, and lipid-lowering medication use.*

*Shading indicates adjusted standardized regression estimates ranging from:*

*
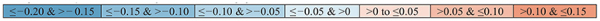
*

### Muscle Area

Among the 24 lipoprotein subfractions that were significantly associated with total muscle area, 21 lipoprotein subfractions were positively associated and three lipoprotein subfractions were inversely associated (Table 2). When examining total muscle area (Figure 1A) by locomotion muscle (Figure 1B) and stabilization muscle (Figure 1C), we found no significant associations between lipoprotein subfractions and locomotion muscle area, whereas 25 lipoprotein subfractions were significantly associated with stabilization muscle area (Supplementary Table 1). When further splitting up the stabilization muscle group, we found no significant associations between lipoprotein subfractions and paraspinal muscle area nor rectus abdominus muscle area, whereas 61 lipoprotein subfractions were significantly associated with oblique muscle area (Supplementary Figures 1C–E and Supplementary Table 1).

### Intermuscular Adipose Tissue Area

Among the 27 lipoprotein subfractions significantly associated with total IMAT area, 20 lipoprotein subfractions were positively associated and seven lipoprotein subfractions were inversely associated (Table 2). When examining total IMAT area (Figure 1G) by muscle groups, we found no significant associations between lipoprotein subfractions and locomotion IMAT area (Figure 1H), whereas 27 lipoprotein subfractions were significantly associated with stabilization IMAT area (Figure 1I). When further splitting up the stabilization muscle group, we found no significant associations between lipoprotein subfractions and paraspinal IMAT area, whereas 29 lipoprotein subfractions were significantly associated with oblique IMAT area and 40 lipoprotein subfractions were associated with rectus abdominis IMAT area (Supplementary Figures 1M–O and Supplementary Table 2).

### Intermuscular Adipose Tissue Density

Among the 39 lipoprotein subfractions significantly associated with total IMAT density, six lipoprotein subfractions were positively associated and 33 lipoprotein subfractions were inversely associated (Table 2). When examining total IMAT density by muscle groups, we found 28 lipoprotein subfractions significantly associated with locomotion IMAT density (Figure 1K) and 33 lipoprotein subfractions associated with stabilization IMAT density (Figure 1L). When further splitting up the stabilization muscle group, we found no significant associations between lipoprotein subfractions and paraspinal IMAT density nor rectus abdominus IMAT density, whereas there were 28 lipoprotein subfractions significantly associated with oblique IMAT density (Supplementary Figures 1R–T and Supplementary Table 3).

### Lipoprotein Subfractions by Content and Density

**FIGURE 2:**
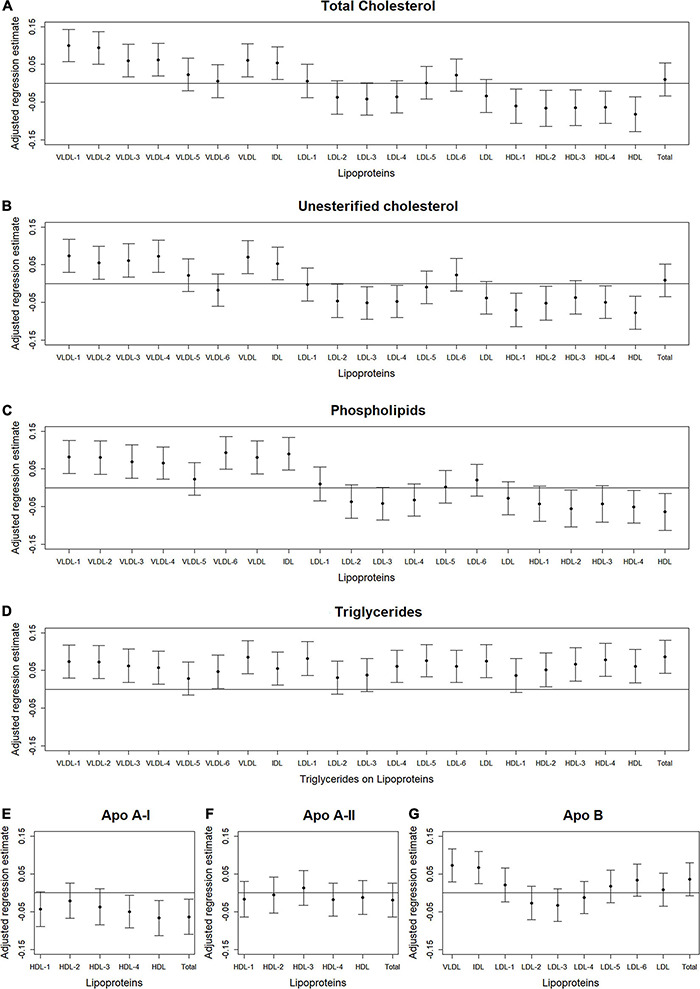
Total abdominal muscle area: Scatterplots of the adjusted associations between 105 lipoprotein subfractions and muscle area ordered from lowest to highest density lipoprotein subfraction and by total cholesterol **(A)**, unesterified cholesterol **(B)**, phospholipids **(C)**, triglycerides **(D)**, apo A-I **(E)**, apo A-II **(F)**, and apo B **(G)** among 947 MESA participants.

**FIGURE 3:**
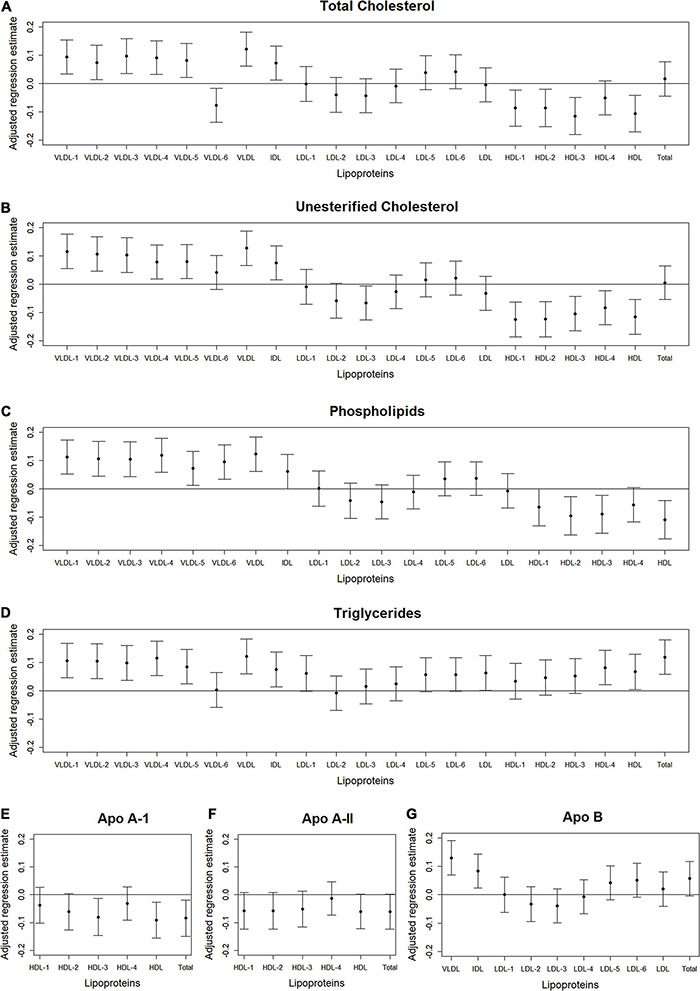
Total abdominal IMAT area: Scatterplots of the adjusted associations between 105 lipoprotein subfractions and IMAT area ordered from lowest to highest density lipoprotein subfraction and by total cholesterol **(A)**, unesterified cholesterol **(B)**, phospholipids **(C)**, triglycerides **(D)**, apo A-I **(E)**, apo A-II **(F)**, and apo B **(G)** among 947 MESA participants.

**FIGURE 4:**
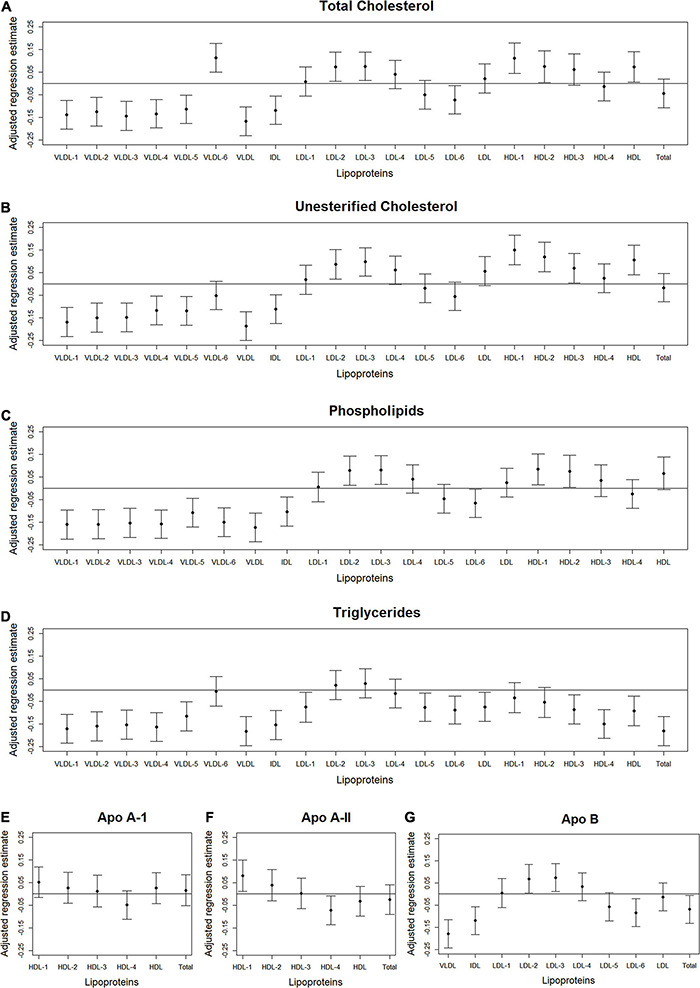
Total abdominal IMAT density: Scatterplots of the adjusted associations between 105 lipoprotein subfractions and IMAT density ordered from lowest to highest density lipoprotein subfraction and by total cholesterol **(A)**, unesterified cholesterol **(B)**, phospholipids **(C)**, triglycerides **(D)**, apo A-I **(E)**, apo A-II **(F)**, and apo B **(G)** among 947 MESA participants.

Figures 2–4 illustrate, by content, the pattern of the associations between lipoprotein subfractions and abdominal body composition as the lipoprotein becomes denser. When examining total cholesterol, unesterified cholesterol, or phospholipids of lipoprotein subfractions, the lowest density lipoprotein subfractions (VLDL-1 to VLDL4) were positively associated with total muscle area (Figures 2A–C) and IMAT area (Figures 3A–C) and inversely associated with IMAT density (Figures 4A–C), and vice versa when examining the higher density lipoprotein subfractions. For the different densities of LDL rich in total cholesterol, unesterified cholesterol, or phospholipids, there tended to be a J-shaped association with muscle area and IMAT area, with the inverse pattern observed for IMAT density, but few associations were significant. Regardless of lipoprotein subfraction density, concentrations of triglycerides in lipoprotein subfractions tended to be positively associated with total muscle area (Figure 2D) and IMAT area (Figure 3D) and tended to be inversely associated with IMAT density (Figure 4D). HDL consisting of apo A-1 tended to be inversely associated with total muscle area (Figure 2E) and IMAT area (Figure 3E), but positively associated with IMAT density (Figure 4E), however, few associations were significant. Similarly, few associations were significant between apo A-II on lipoprotein subfractions and abdominal body composition (Figures 2F, 3F, 4F). For apo B, there appeared to be a J-shaped pattern in associations between lipoprotein subfractions and muscle area (Figure 2G) and IMAT area (Figure 3G), with the inverse pattern for IMAT density (Figure 4G).

## Discussion

Higher levels of total cholesterol, unesterified cholesterol, phospholipids, triglycerides, and apo B in several VLDL subclasses and lower levels of total and unesterified cholesterol in HDL subclasses were associated with higher total abdominal muscle area and IMAT area, but lower IMAT density. Conversely, we found no significant associations between lipoprotein subfractions and total abdominal muscle density. Significant associations between lipoprotein subfractions and total muscle area *and* IMAT area were mostly statistically driven by abdominal stabilization muscles, particularly by the oblique muscles, while associations between lipoprotein subfractions and IMAT density were influenced by both locomotion (psoas) and stabilization muscles, with the strongest associations observed in the oblique muscles. The aforementioned associations did not differ significantly by gender, race/ethnicity, or height.

We found several VLDL subfractions associated with abdominal body composition. VLDL are synthesized in the liver (Feingold, 2021), with the main function of transporting endogenous lipids from the liver, through the blood, to muscle and adipose tissue (Nelson and Cox, 2017), where free fatty acids within the VLDL particles are then released. Once removed, fatty acids can be oxidized by myocytes and used as an energy source during fasting states or reconverted to triglycerides by adipocytes and stored in intracellular lipid droplets when insulin is high (e.g., after a meal) (Nelson and Cox, 2017). However, with aging and excess caloric consumption, fat can start to accumulate ectopically instead, including in and around skeletal muscle (Miljkovic and Zmuda, 2010). Since VLDL are rich with triglycerides, overproduction or inefficient clearance of VLDL can result in hypertriglyceridemia (Lee and Lin, 2020). Our results demonstrated that higher levels of circulating triglycerides, total cholesterol, unesterified cholesterol, phospholipids, and apo B in VLDL subclasses were associated with higher muscle area and IMAT area, but lower IMAT density. Lower IMAT density (i.e., more negative HU) indicates worse quality adipose tissue beneath the fascia and between muscle groups. This pattern of adipose tissue indicates a higher lipid content per adipocyte and lower vascularity (Rosenquist et al., 2015). Thus, the identified VLDL subfractions may indicate a metabolic profile contributing to accumulation of IMAT that is made up of particularly worse quality adipose tissue. However, causality between VLDL subfractions and IMAT cannot be determined from this report.

We also found total and unesterified cholesterol in HDL subclasses associated with abdominal body composition. HDL is a unique lipoprotein because it can remove cholesterol from cells, including from foam cells of atherosclerotic plaques, where they are transported to the liver for catabolism and excretion (Nelson and Cox, 2017). In this report, we observed higher HDL cholesterol and HDL unesterified cholesterol were associated with lower muscle area, lower IMAT area, and higher IMAT density (i.e., better quality adipose tissue around the muscle). Thus, these HDL subclasses may be removing fats that have accumulated around the muscle and preventing additional fats from accumulating, resulting in less IMAT with a lower lipid content per adipocyte. However, it remains to be determined whether HDL plays a direct role in the health and composition of skeletal muscle.

Associations between lipoprotein subfractions and abdominal body composition were strongest among the stabilization muscle group in this cohort. The majority of abdominal stabilization muscles are composed of type I muscle fibers, i.e., slow-twitch fibers (Häggmark and Thorstensson, 1979). Whereas the majority of abdominal locomotion (psoas) muscles are composed of type II muscle fibers, i.e., fast-twitch fibers (Arbanas et al., 2009). Type I muscle fibers have a greater fat content than type II muscle fibers (He et al., 2001), potentially explaining why muscles of stabilization were driving associations between circulating lipoprotein subfractions and overall abdominal body composition.

When examining each individual muscle subgroup, we consistently found the oblique muscles were most strongly associated with lipoprotein subfractions. Studies documenting fiber type among the oblique muscles are rare. One study found that the majority of oblique muscles were composed of type I muscle fibers (Häggmark and Thorstensson, 1979). However, the study also observed much larger differences in fiber composition between individuals than between muscle groups. It may be that the oblique muscles are the most relevant in this report since they are the largest abdominal muscle or it may be that these muscles are truly biologically operative, warranting further investigation. These findings indicate the importance of examining quantity and quality of each abdominal muscle group separately.

We found higher levels of several VLDL contents and lower levels of HDL contents associated with higher total abdominal muscle area. Similarly, previous reports have found higher muscle area to be associated with worse health outcomes. For example, higher abdominal muscle area was associated with higher coronary artery calcium among the MESA cohort (Crawford et al., 2020), higher risk of incident diabetes among overweight and obese older women from the Health, Aging, and Body Composition Study (Larsen et al., 2016), and higher risk of all-cause mortality among MESA men (Larsen et al., 2020). These associations with total abdominal muscle area may seem counterintuitive, but higher muscle area does not necessarily indicate healthier muscle since muscle area could be larger due to a larger amount of worse quality muscle, i.e., lower muscle density with greater fat infiltration. In this report, the only information on muscle quality was from CT-derived muscle density, which is interpreted as a less dense muscle indicating worse quality muscle with greater fat infiltration (Goodpaster et al., 2000). However, intramuscular adipose tissue is only one component of muscle quality and does not address the muscle’s ability to maintain healthy metabolism and neural activity, force production, and architecture and composition (such as extent of fibrosis). Future reports with a better measure of muscle density will be needed to further understand these associations between lipoprotein subfractions and higher muscle area.

As stated above, a limitation of the current report was only having information on muscle quality from CT-derived muscle density, which is interpreted as worse quality muscle with greater fat infiltration (Goodpaster et al., 2000). If we had a more complete measure of muscle quality that also incorporated the muscle’s ability to maintain healthy metabolism and neural activity, force production, and architecture and composition (e.g., extent of fibrosis), then we may have been more successful at identifying significant associations between lipoprotein subfractions and muscle quality. Another limitation was the lack of power to examine differences in associations between lipoprotein subfractions and abdominal body composition by race and sex. Despite these limitations, our report had several strengths, including the large, well-characterized diverse U.S. cohort, multiple measures of abdominal body composition, including area and density of total muscle and IMAT derived from computed tomography scans, as well as the composition of four individual abdominal muscle groups, which were used to determine if a specific muscle group was driving significant associations. Other strengths included the use of frozen serum samples that had never been thawed to measure a wide range of lipoprotein subclasses.

Several lipoprotein subclasses were associated with overall abdominal muscle area, IMAT area, and IMAT density, with associations mostly statistically driven by abdominal stabilization muscles, particularly by the oblique muscle subgroup. This illustrates the importance of examining quality and quantity of each muscle group separately. Future work is needed to determine whether the observed associations indicate a lipoprotein profile that contributes to worse skeletal muscle with fat infiltration among adults ages 44–84 years.

## Data Availability Statement

The raw data supporting the conclusions of this article will be made available by the authors, without undue reservation.

## Ethics Statement

The studies involving human participants were reviewed and approved by the MESA and its ancillary studies were approved by the Institutional Review Board of each participating site. The patients/participants provided their written informed consent to participate in this study.

## Author Contributions

DH: MESA study lipoprotein ancillary study. IM and MM: manuscript concept. MM: data analysis and manuscript writing. All authors interpretation of data and manuscript editing and critical review.

## Conflict of Interest

The authors declare that the research was conducted in the absence of any commercial or financial relationships that could be construed as a potential conflict of interest.

## Publisher’s Note

All claims expressed in this article are solely those of the authors and do not necessarily represent those of their affiliated organizations, or those of the publisher, the editors and the reviewers. Any product that may be evaluated in this article, or claim that may be made by its manufacturer, is not guaranteed or endorsed by the publisher.
