# Supplementary material for: Associations Between Lipoprotein Subfractions and Area and Density of Abdominal Muscle and Intermuscular Adipose Tissue: The Multi-Ethnic Study of Atherosclerosis
Source: Front Physiol. 2021 Sep 27;12:713048. doi: 10.3389/fphys.2021.713048 (PMC8502976; doi:10.3389/fphys.2021.713048)
Supplement: Supplementary file 1 [file Data_Sheet_1.docx]

Supplementary Material

# Supplementary Figures and Tables

## Supplementary Figures


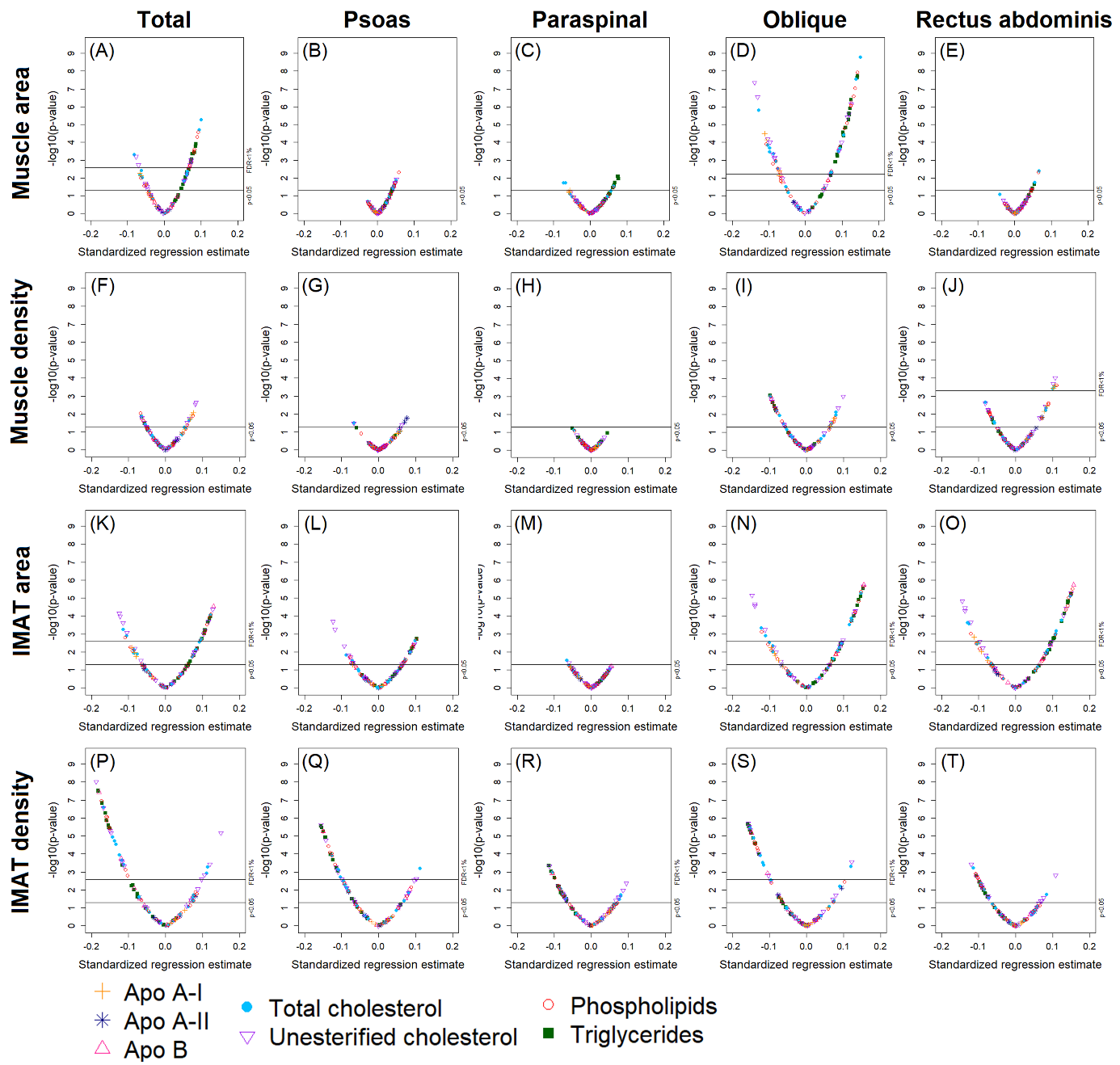


**Supplementary Figure 1.** Volcano plots of the adjusted associations* between 105 lipoprotein subfractions and area and density of muscle and IMAT of the total abdominal, psoas, paraspinal, oblique, and rectus abdominus muscles among 947 MESA participants.

*Associations were adjusted for age, gender, race, alternate healthy eating index, moderate/vigorous physical activity, sedentary behavior, and lipid-lowering medication use.

## Supplementary Tables

**Supplementary Table 1.** Adjusted standardized regression estimates of associations between 105 lipoprotein subfractions and **muscle area** of the total abdomen and locomotion (psoas), stabilization (paraspinal, oblique, and rectus abdominus), and individual muscle groups.

| **Lipoprotein** **subfractions:** | **Abdominal muscle area** | | | | | | |
| --- | --- | --- | --- | --- | --- | --- | --- |
|  | **Total** | **Locomotion (psoas)** | **Stabilization*** | **Paraspinal** | **Oblique** | **Rectus abdominis** |  |
| Total Cholesterol: |  |  |  |  |  |  |  |
| VLDL-1 | 0.10 (0.02), p<.0001, q=.0006 | 0.03 (0.02), p=.11, q=.43 | 0.11 (0.02), p<.0001, q=.0002 | 0.06 (0.03), p=.03, q=.38 | 0.15 (0.02), p<.0001, q<.0001 | 0.07 (0.02), p=.004, q=.25 |  |
| VLDL-2 | 0.09 (0.02), p<.0001, q=.001 | 0.04 (0.02), p=.03, q=.25 | 0.10 (0.02), p<.0001, q=.0007 | 0.06 (0.03), p=.05, q=.46 | 0.14 (0.02), p<.0001, q<.0001 | 0.06 (0.02), p=.02, q=.48 |  |
| VLDL-3 | 0.06 (0.02), p=.007, q=.02 | 0.05 (0.02), p=.01, q=.25 | 0.06 (0.02), p=.02, q=.04 | 0.02 (0.03), p=.51, q=.74 | 0.09 (0.03), p=.0002, q=.0006 | 0.03 (0.02), p=.23, q=.78 |  |
| VLDL-4 | 0.06 (0.02), p=.004, q=.02 | 0.04 (0.02), p=.03, q=.25 | 0.06 (0.02), p=.008, q=.02 | 0.03 (0.03), p=.24, q=.61 | 0.09 (0.02), p=.0003, q=.0008 | 0.03 (0.02), p=.25, q=.78 |  |
| VLDL-5 | 0.02 (0.02), p=.29, q=.37 | -0.01 (0.02), p=.49, q=.88 | 0.03 (0.02), p=.16, q=.22 | 0.00003 (0.03), p=.999, q=.999 | 0.07 (0.02), p=.005, q=.009 | 0.008 (0.02), p=.72, q=.92 |  |
| VLDL-6 | 0.005 (0.02), p=.81, q=.84 | 0.004 (0.02), p=.85, q=.96 | 0.005 (0.02), p=.83, q=.87 | 0.05 (0.03), p=.11, q=.49 | -0.02 (0.03), p=.38, q=.43 | -0.04 (0.02), p=.08, q=.65 |  |
| VLDL | 0.06 (0.02), p=.006, q=.02 | 0.04 (0.02), p=.04, q=.25 | 0.06 (0.02), p=.01, q=.03 | 0.01 (0.03), p=.62, q=.81 | 0.10 (0.02), p<.0001, q=.0001 | 0.03 (0.02), p=.15, q=.75 |  |
| IDL | 0.05 (0.02), p=.01, q=.03 | 0.04 (0.02), p=.03, q=.25 | 0.05 (0.02), p=.03, q=.06 | 0.03 (0.03), p=.32, q=.65 | 0.07 (0.02), p=.005, q=.009 | 0.03 (0.02), p=.22, q=.78 |  |
| LDL-1 | 0.006 (0.02), p=.79, q=.83 | 0.02 (0.02), p=.25, q=.69 | -0.0004 (0.03), p=.99, q=.99 | -0.001 (0.03), p=.97, q=.998 | -0.01 (0.03), p=.65, q=.68 | 0.03 (0.02), p=.22, q=.78 |  |
| LDL-2 | -0.04 (0.02), p=.10, q=.16 | 0.003 (0.02), p=.87, q=.96 | -0.05 (0.02), p=.05, q=.09 | -0.03 (0.03), p=.29, q=.63 | -0.08 (0.03), p=.003, q=.006 | 0.02 (0.02), p=.47, q=.84 |  |
| LDL-3 | -0.04 (0.02), p=.06, q=.10 | 0.007 (0.02), p=.73, q=.96 | -0.05 (0.02), p=.02, q=.05 | -0.04 (0.03), p=.22, q=.61 | -0.08 (0.02), p=.001, q=.003 | 0.004 (0.02), p=.86, q=.97 |  |
| LDL-4 | -0.04 (0.02), p=.10, q=.16 | -0.01 (0.02), p=.59, q=.94 | -0.04 (0.02), p=.09, q=.14 | -0.03 (0.03), p=.38, q=.66 | -0.05 (0.02), p=.03, q=.04 | -0.02 (0.02), p=.45, q=.84 |  |
| LDL-5 | 0.001 (0.02), p=.96, q=.96 | -0.02 (0.02), p=.40, q=.84 | 0.007 (0.02), p=.77, q=.82 | -0.003 (0.03), p=.91, q=.99 | 0.03 (0.02), p=.28, q=.33 | -0.02 (0.02), p=.39, q=.84 |  |
| LDL-6 | 0.02 (0.02), p=.33, q=.41 | -0.003 (0.02), p=.90, q=.96 | 0.03 (0.02), p=.25, q=.31 | 0.002 (0.03), p=.94, q=.99 | 0.05 (0.02), p=.04, q=.05 | 0.01 (0.02), p=.59, q=.85 |  |
| LDL | -0.03 (0.02), p=.13, q=.19 | -0.002 (0.02), p=.94, q=.97 | -0.04 (0.02), p=.09, q=.14 | -0.04 (0.03), p=.18, q=.59 | -0.05 (0.03), p=.06, q=.08 | 0.002 (0.02), p=.95, q=.99 |  |
| HDL-1 | -0.06 (0.02), p=.009, q=.02 | -0.03 (0.02), p=.21, q=.69 | -0.07 (0.03), p=.009, q=.03 | -0.04 (0.03), p=.25, q=.61 | -0.10 (0.03), p=.0002, q=.0006 | -0.02 (0.02), p=.48, q=.84 |  |
| HDL-2 | -0.07 (0.02), p=.007, q=.02 | -0.01 (0.02), p=.55, q=.90 | -0.08 (0.03), p=.004, q=.01 | -0.06 (0.03), p=.08, q=.46 | -0.10 (0.03), p=.0003, q=.0008 | -0.02 (0.03), p=.44, q=.84 |  |
| HDL-3 | -0.06 (0.02), p=.007, q=.02 | -0.02 (0.02), p=.49, q=.88 | -0.08 (0.03), p=.004, q=.01 | -0.05 (0.03), p=.11, q=.49 | -0.10 (0.03), p=.0001, q=.0004 | -0.01 (0.03), p=.57, q=.85 |  |
| HDL-4 | -0.06 (0.02), p=.004, q=.01 | -0.01 (0.02), p=.48, q=.88 | -0.07 (0.02), p=.002, q=.009 | -0.07 (0.03), p=.02, q=.38 | -0.09 (0.02), p=.0004, q=.001 | 0.001 (0.02), p=.97, q=.995 |  |
| HDL | -0.08 (0.02), p=.0005, q=.004 | -0.02 (0.02), p=.47, q=.88 | -0.10 (0.03), p=.0002, q=.002 | -0.07 (0.03), p=.02, q=.38 | -0.13 (0.03), p<.0001, q<.0001 | -0.008 (0.02), p=.74, q=.92 |  |
| Total | 0.01 (0.02), p=.64, q=.71 | 0.02 (0.02), p=.25, q=.69 | 0.005 (0.02), p=.83, q=.87 | -0.01 (0.03), p=.64, q=.81 | 0.01 (0.03), p=.64, q=.68 | 0.03 (0.02), p=.15, q=.75 |  |
| Unesterified Cholesterol: |  |  |  |  |  |  |  |
| VLDL-1 | 0.07 (0.02), p=.001, q=.006 | 0.05 (0.02), p=.02, q=.25 | 0.08 (0.02), p=.002, q=.009 | 0.02 (0.03), p=.48, q=.71 | 0.12 (0.03), p<.0001, q<.0001 | 0.04 (0.02), p=.12, q=.68 |  |
| VLDL-2 | 0.06 (0.02), p=.01, q=.03 | 0.05 (0.02), p=.02, q=.25 | 0.05 (0.02), p=.03, q=.06 | 0.01 (0.03), p=.72, q=.86 | 0.09 (0.03), p=.0003, q=.0008 | 0.03 (0.02), p=.25, q=.78 |  |
| VLDL-3 | 0.06 (0.02), p=.006, q=.02 | 0.05 (0.02), p=.01, q=.25 | 0.06 (0.02), p=.02, q=.04 | 0.02 (0.03), p=.56, q=.78 | 0.10 (0.03), p=.0001, q=.0003 | 0.03 (0.02), p=.28, q=.78 |  |
| VLDL-4 | 0.07 (0.02), p=.001, q=.006 | 0.03 (0.02), p=.09, q=.37 | 0.08 (0.02), p=.001, q=.006 | 0.04 (0.03), p=.18, q=.59 | 0.11 (0.02), p<.0001, q<.0001 | 0.03 (0.02), p=.16, q=.78 |  |
| VLDL-5 | 0.02 (0.02), p=.32, q=.40 | -0.01 (0.02), p=.59, q=.94 | 0.03 (0.02), p=.20, q=.26 | 0.008 (0.03), p=.78, q=.92 | 0.06 (0.02), p=.03, q=.04 | 0.008 (0.02), p=.72, q=.92 |  |
| VLDL-6 | -0.02 (0.02), p=.41, q=.49 | -0.002 (0.02), p=.91, q=.96 | -0.02 (0.02), p=.37, q=.43 | -0.03 (0.03), p=.37, q=.66 | -0.03 (0.02), p=.26, q=.30 | 0.02 (0.02), p=.35, q=.84 |  |
| VLDL | 0.07 (0.02), p=.002, q=.009 | 0.04 (0.02), p=.06, q=.32 | 0.07 (0.02), p=.003, q=.01 | 0.02 (0.03), p=.50, q=.73 | 0.12 (0.03), p<.0001, q<.0001 | 0.05 (0.02), p=.05, q=.48 |  |
| IDL | 0.05 (0.02), p=.02, q=.03 | 0.04 (0.02), p=.04, q=.25 | 0.05 (0.02), p=.03, q=.06 | 0.03 (0.03), p=.24, q=.61 | 0.07 (0.02), p=.007, q=.01 | 0.02 (0.02), p=.31, q=.80 |  |
| LDL-1 | -0.002 (0.02), p=.91, q=.92 | 0.01 (0.02), p=.54, q=.90 | -0.007 (0.02), p=.77, q=.82 | -0.002 (0.03), p=.95, q=.99 | -0.02 (0.03), p=.46, q=.51 | 0.01 (0.02), p=.58, q=.85 |  |
| LDL-2 | -0.05 (0.02), p=.04, q=.08 | 0.004 (0.02), p=.86, q=.96 | -0.06 (0.02), p=.02, q=.04 | -0.04 (0.03), p=.20, q=.59 | -0.09 (0.03), p=.0005, q=.001 | 0.01 (0.02), p=.58, q=.85 |  |
| LDL-3 | -0.05 (0.02), p=.02, q=.04 | 0.005 (0.02), p=.80, q=.96 | -0.07 (0.02), p=.007, q=.02 | -0.04 (0.03), p=.17, q=.59 | -0.10 (0.02), p<.0001, q=.0003 | -0.003 (0.02), p=.89, q=.97 |  |
| LDL-4 | -0.05 (0.02), p=.03, q=.06 | -0.02 (0.02), p=.42, q=.86 | -0.05 (0.02), p=.03, q=.05 | -0.03 (0.03), p=.28, q=.62 | -0.07 (0.02), p=.003, q=.006 | -0.02 (0.02), p=.34, q=.84 |  |
| LDL-5 | -0.01 (0.02), p=.66, q=.71 | -0.02 (0.02), p=.22, q=.69 | -0.004 (0.02), p=.87, q=.90 | -0.005 (0.03), p=.87, q=.98 | 0.009 (0.02), p=.71, q=.74 | -0.03 (0.02), p=.18, q=.78 |  |
| LDL-6 | 0.02 (0.02), p=.28, q=.37 | 0.006 (0.02), p=.78, q=.96 | 0.03 (0.02), p=.25, q=.31 | 0.003 (0.03), p=.91, q=.99 | 0.05 (0.02), p=.05, q=.06 | 0.01 (0.02), p=.52, q=.84 |  |
| Unesterified Cholesterol cont.: |  |  |  |  |  |  |  |
| LDL | -0.04 (0.02), p=.09, q=.15 | -0.002 (0.02), p=.90, q=.96 | -0.05 (0.02), p=.06, q=.09 | -0.03 (0.03), p=.27, q=.61 | -0.06 (0.02), p=.01, q=.02 | -0.005 (0.02), p=.82, q=.97 |  |
| HDL-1 | -0.07 (0.02), p=.002, q=.009 | -0.006 (0.02), p=.77, q=.96 | -0.09 (0.02), p=.0006, q=.004 | -0.04 (0.03), p=.19, q=.59 | -0.13 (0.03), p<.0001, q<.0001 | -0.03 (0.02), p=.28, q=.78 |  |
| HDL-2 | -0.05 (0.02), p=.02, q=.05 | 0.001 (0.02), p=.94, q=.97 | -0.07 (0.03), p=.01, q=.03 | -0.02 (0.03), p=.42, q=.68 | -0.10 (0.03), p<.0001, q=.0002 | -0.03 (0.02), p=.27, q=.78 |  |
| HDL-3 | -0.04 (0.02), p=.10, q=.16 | 0.02 (0.02), p=.40, q=.84 | -0.05 (0.02), p=.04, q=.07 | -0.02 (0.03), p=.45, q=.69 | -0.09 (0.03), p=.0008, q=.002 | -0.003 (0.02), p=.89, q=.97 |  |
| HDL-4 | -0.05 (0.02), p=.03, q=.05 | 0.003 (0.02), p=.89, q=.96 | -0.06 (0.02), p=.01, q=.03 | -0.05 (0.03), p=.12, q=.49 | -0.08 (0.02), p=.0007, q=.002 | -0.003 (0.02), p=.89, q=.97 |  |
| HDL | -0.08 (0.02), p=.0006, q=.005 | -0.006 (0.02), p=.76, q=.96 | -0.09 (0.02), p=.0001, q=.002 | -0.05 (0.03), p=.07, q=.46 | -0.14 (0.03), p<.0001, q<.0001 | -0.01 (0.02), p=.60, q=.85 |  |
| Total | 0.009 (0.02), p=.69, q=.74 | 0.03 (0.02), p=.17, q=.63 | 0.002 (0.02), p=.94, q=.95 | -0.003 (0.03), p=.90, q=.99 | -0.002 (0.02), p=.93, q=.93 | 0.02 (0.02), p=.31, q=.80 |  |
| Phospholipids: |  |  |  |  |  |  |  |
| VLDL-1 | 0.08 (0.02), p=.0003, q=.004 | 0.06 (0.02), p=.005, q=.25 | 0.08 (0.02), p=.0008, q=.005 | 0.03 (0.03), p=.27, q=.61 | 0.12 (0.02), p<.0001, q<.0001 | 0.04 (0.02), p=.05, q=.48 |  |
| VLDL-2 | 0.08 (0.02), p=.0004, q=.004 | 0.05 (0.02), p=.03, q=.25 | 0.09 (0.02), p=.0006, q=.004 | 0.03 (0.03), p=.32, q=.65 | 0.13 (0.03), p<.0001, q<.0001 | 0.05 (0.02), p=.04, q=.48 |  |
| VLDL-3 | 0.07 (0.02), p=.002, q=.009 | 0.05 (0.02), p=.02, q=.25 | 0.07 (0.02), p=.004, q=.01 | 0.02 (0.03), p=.45, q=.69 | 0.11 (0.03), p<.0001, q<.0001 | 0.03 (0.02), p=.15, q=.75 |  |
| VLDL-4 | 0.07 (0.02), p=.003, q=.01 | 0.04 (0.02), p=.06, q=.32 | 0.07 (0.02), p=.004, q=.01 | 0.03 (0.03), p=.34, q=.65 | 0.11 (0.02), p<.0001, q<.0001 | 0.03 (0.02), p=.22, q=.78 |  |
| VLDL-5 | 0.02 (0.02), p=.29, q=.37 | 0.004 (0.02), p=.83, q=.96 | 0.03 (0.02), p=.25, q=.31 | -0.007 (0.03), p=.82, q=.94 | 0.06 (0.02), p=.01, q=.02 | 0.01 (0.02), p=.52, q=.84 |  |
| VLDL-6 | 0.09 (0.02), p<.0001, q=.001 | 0.04 (0.02), p=.08, q=.37 | 0.10 (0.02), p<.0001, q=.0007 | 0.05 (0.03), p=.08, q=.46 | 0.14 (0.02), p<.0001, q<.0001 | 0.07 (0.02), p=.005, q=.25 |  |
| VLDL | 0.08 (0.02), p=.0003, q=.004 | 0.04 (0.02), p=.09, q=.37 | 0.09 (0.02), p=.0003, q=.003 | 0.03 (0.03), p=.24, q=.61 | 0.13 (0.02), p<.0001, q<.0001 | 0.05 (0.02), p=.05, q=.48 |  |
| IDL | 0.09 (0.02), p<.0001, q=.001 | 0.05 (0.02), p=.03, q=.25 | 0.10 (0.02), p<.0001, q=.001 | 0.06 (0.03), p=.03, q=.38 | 0.12 (0.02), p<.0001, q<.0001 | 0.05 (0.02), p=.05, q=.48 |  |
| LDL-1 | 0.01 (0.02), p=.66, q=.71 | 0.02 (0.02), p=.34, q=.82 | 0.006 (0.03), p=.81, q=.86 | 0.006 (0.03), p=.83, q=.95 | -0.005 (0.03), p=.85, q=.85 | 0.03 (0.02), p=.25, q=.78 |  |
| LDL-2 | -0.04 (0.02), p=.11, q=.16 | 0.004 (0.02), p=.86, q=.96 | -0.05 (0.03), p=.06, q=.10 | -0.03 (0.03), p=.32, q=.65 | -0.08 (0.03), p=.003, q=.006 | 0.02 (0.02), p=.48, q=.84 |  |
| LDL-3 | -0.04 (0.02), p=.06, q=.10 | 0.004 (0.02), p=.83, q=.96 | -0.05 (0.02), p=.03, q=.05 | -0.03 (0.03), p=.26, q=.61 | -0.08 (0.02), p=.001, q=.002 | 0.0004 (0.02), p=.98, q=.995 |  |
| LDL-4 | -0.03 (0.02), p=.14, q=.20 | -0.01 (0.02), p=.50, q=.88 | -0.04 (0.02), p=.14, q=.19 | -0.02 (0.03), p=.55, q=.78 | -0.05 (0.02), p=.04, q=.06 | -0.02 (0.02), p=.38, q=.84 |  |
| LDL-5 | 0.002 (0.02), p=.91, q=.92 | -0.02 (0.02), p=.38, q=.84 | 0.009 (0.02), p=.71, q=.78 | -0.0004 (0.03), p=.99, q=.999 | 0.03 (0.02), p=.24, q=.29 | -0.02 (0.02), p=.31, q=.80 |  |
| LDL-6 | 0.02 (0.02), p=.36, q=.44 | -0.005 (0.02), p=.80, q=.96 | 0.03 (0.02), p=.26, q=.32 | 0.002 (0.03), p=.96, q=.99 | 0.05 (0.02), p=.04, q=.05 | 0.01 (0.02), p=.63, q=.88 |  |
| LDL | -0.03 (0.02), p=.21, q=.28 | -0.005 (0.02), p=.81, q=.96 | -0.03 (0.02), p=.17, q=.23 | -0.03 (0.03), p=.32, q=.65 | -0.04 (0.03), p=.11, q=.13 | 0.0004 (0.02), p=.99, q=.995 |  |
| HDL-1 | -0.04 (0.02), p=.07, q=.13 | -0.03 (0.02), p=.23, q=.69 | -0.04 (0.03), p=.09, q=.14 | -0.02 (0.03), p=.56, q=.78 | -0.07 (0.03), p=.01, q=.02 | -0.02 (0.02), p=.49, q=.84 |  |
| HDL-2 | -0.06 (0.02), p=.03, q=.05 | -0.02 (0.02), p=.29, q=.73 | -0.06 (0.03), p=.03, q=.05 | -0.03 (0.03), p=.39, q=.67 | -0.09 (0.03), p=.002, q=.003 | -0.03 (0.03), p=.28, q=.78 |  |
| HDL-3 | -0.04 (0.02), p=.08, q=.14 | -0.02 (0.02), p=.34, q=.82 | -0.05 (0.03), p=.09, q=.14 | -0.02 (0.03), p=.44, q=.69 | -0.07 (0.03), p=.01, q=.02 | -0.01 (0.03), p=.67, q=.88 |  |
| HDL-4 | -0.05 (0.02), p=.02, q=.05 | -0.02 (0.02), p=.30, q=.76 | -0.06 (0.02), p=.02, q=.05 | -0.05 (0.03), p=.12, q=.49 | -0.07 (0.03), p=.005, q=.009 | 0.001 (0.02), p=.97, q=.995 |  |
| HDL | -0.06 (0.02), p=.01, q=.03 | -0.02 (0.02), p=.43, q=.86 | -0.07 (0.03), p=.007, q=.02 | -0.04 (0.03), p=.20, q=.59 | -0.11 (0.03), p=.0001, q=.0004 | -0.01 (0.03), p=.60, q=.85 |  |
| Triglycerides: |  |  |  |  |  |  |  |
| VLDL-1 | 0.07 (0.02), p=.001, q=.006 | 0.04 (0.02), p=.04, q=.28 | 0.08 (0.02), p=.002, q=.008 | 0.03 (0.03), p=.38, q=.66 | 0.12 (0.03), p<.0001, q<.0001 | 0.05 (0.02), p=.04, q=.48 |  |
| VLDL-2 | 0.07 (0.02), p=.001, q=.007 | 0.04 (0.02), p=.03, q=.25 | 0.08 (0.02), p=.002, q=.01 | 0.02 (0.03), p=.45, q=.69 | 0.12 (0.03), p<.0001, q<.0001 | 0.05 (0.02), p=.06, q=.48 |  |
| VLDL-3 | 0.06 (0.02), p=.006, q=.02 | 0.05 (0.02), p=.03, q=.25 | 0.06 (0.02), p=.01, q=.03 | 0.01 (0.03), p=.64, q=.81 | 0.10 (0.03), p<.0001, q=.0002 | 0.04 (0.02), p=.09, q=.65 |  |
| VLDL-4 | 0.06 (0.02), p=.009, q=.02 | 0.03 (0.02), p=.11, q=.44 | 0.06 (0.02), p=.01, q=.03 | 0.01 (0.03), p=.62, q=.81 | 0.10 (0.02), p<.0001, q=.0001 | 0.03 (0.02), p=.19, q=.78 |  |
| VLDL-5 | 0.03 (0.02), p=.20, q=.27 | -0.005 (0.02), p=.79, q=.96 | 0.04 (0.02), p=.13, q=.18 | 0.004 (0.03), p=.90, q=.99 | 0.07 (0.03), p=.005, q=.009 | 0.01 (0.02), p=.54, q=.84 |  |
| VLDL-6 | 0.05 (0.02), p=.04, q=.07 | 0.01 (0.02), p=.48, q=.88 | 0.05 (0.02), p=.03, q=.06 | 0.06 (0.03), p=.03, q=.38 | 0.04 (0.03), p=.10, q=.13 | 0.01 (0.02), p=.64, q=.88 |  |
| VLDL | 0.08 (0.02), p=.0002, q=.003 | 0.04 (0.02), p=.06, q=.32 | 0.09 (0.02), p=.0002, q=.002 | 0.03 (0.03), p=.24, q=.61 | 0.14 (0.03), p<.0001, q<.0001 | 0.05 (0.02), p=.04, q=.48 |  |
| IDL | 0.06 (0.02), p=.01, q=.03 | 0.04 (0.02), p=.06, q=.32 | 0.06 (0.02), p=.02, q=.05 | 0.03 (0.03), p=.35, q=.65 | 0.08 (0.03), p=.001, q=.003 | 0.02 (0.02), p=.38, q=.84 |  |
| LDL-1 | 0.08 (0.02), p=.0004, q=.004 | 0.004 (0.02), p=.84, q=.96 | 0.10 (0.03), p<.0001, q=.001 | 0.08 (0.03), p=.01, q=.38 | 0.12 (0.03), p<.0001, q<.0001 | 0.04 (0.02), p=.11, q=.68 |  |
| LDL-2 | 0.03 (0.02), p=.17, q=.23 | 0.03 (0.02), p=.20, q=.69 | 0.03 (0.02), p=.22, q=.29 | 0.04 (0.03), p=.22, q=.61 | 0.02 (0.03), p=.45, q=.50 | 0.02 (0.02), p=.51, q=.84 |  |
| LDL-3 | 0.04 (0.02), p=.09, q=.15 | -0.004 (0.02), p=.83, q=.96 | 0.05 (0.02), p=.05, q=.08 | 0.06 (0.03), p=.04, q=.38 | 0.04 (0.03), p=.11, q=.14 | -0.004 (0.02), p=.85, q=.97 |  |
| LDL-4 | 0.06 (0.02), p=.005, q=.02 | 0.0004 (0.02), p=.99, q=.99 | 0.08 (0.02), p=.001, q=.008 | 0.08 (0.03), p=.008, q=.38 | 0.09 (0.02), p=.0005, q=.001 | -0.007 (0.02), p=.77, q=.93 |  |
| LDL-5 | 0.08 (0.02), p=.0004, q=.004 | 0.008 (0.02), p=.71, q=.96 | 0.09 (0.02), p=.0001, q=.002 | 0.07 (0.03), p=.02, q=.38 | 0.12 (0.02), p<.0001, q<.0001 | 0.007 (0.02), p=.75, q=.93 |  |
| LDL-6 | 0.06 (0.02), p=.005, q=.02 | 0.007 (0.02), p=.71, q=.96 | 0.07 (0.02), p=.002, q=.009 | 0.04 (0.03), p=.15, q=.58 | 0.10 (0.02), p<.0001, q=.0001 | 0.03 (0.02), p=.20, q=.78 |  |
| LDL | 0.07 (0.02), p=.0009, q=.006 | 0.02 (0.02), p=.43, q=.86 | 0.09 (0.02), p=.0004, q=.003 | 0.07 (0.03), p=.03, q=.38 | 0.11 (0.03), p<.0001, q<.0001 | 0.02 (0.02), p=.40, q=.84 |  |
| Triglycerides cont.: |  |  |  |  |  |  |  |
| HDL-1 | 0.04 (0.02), p=.11, q=.17 | 0.02 (0.02), p=.35, q=.82 | 0.04 (0.03), p=.12, q=.18 | 0.03 (0.03), p=.27, q=.61 | 0.05 (0.03), p=.08, q=.11 | 0.007 (0.02), p=.76, q=.93 |  |
| HDL-2 | 0.05 (0.02), p=.02, q=.05 | 0.03 (0.02), p=.23, q=.69 | 0.06 (0.03), p=.03, q=.05 | 0.04 (0.03), p=.15, q=.58 | 0.07 (0.03), p=.007, q=.01 | 0.01 (0.02), p=.66, q=.88 |  |
| HDL-3 | 0.07 (0.02), p=.003, q=.01 | 0.02 (0.02), p=.26, q=.69 | 0.08 (0.02), p=.003, q=.01 | 0.05 (0.03), p=.07, q=.46 | 0.10 (0.03), p=.0002, q=.0005 | 0.02 (0.02), p=.42, q=.84 |  |
| HDL-4 | 0.08 (0.02), p=.0004, q=.004 | 0.02 (0.02), p=.23, q=.69 | 0.09 (0.02), p=.0003, q=.003 | 0.05 (0.03), p=.08, q=.46 | 0.12 (0.02), p<.0001, q<.0001 | 0.04 (0.02), p=.10, q=.66 |  |
| HDL | 0.06 (0.02), p=.007, q=.02 | 0.02 (0.02), p=.28, q=.73 | 0.07 (0.03), p=.006, q=.02 | 0.05 (0.03), p=.10, q=.49 | 0.09 (0.03), p=.0006, q=.001 | 0.02 (0.02), p=.40, q=.84 |  |
| Total | 0.09 (0.02), p=.0001, q=.003 | 0.04 (0.02), p=.08, q=.37 | 0.10 (0.02), p=.0001, q=.002 | 0.04 (0.03), p=.19, q=.59 | 0.14 (0.03), p<.0001, q<.0001 | 0.05 (0.02), p=.02, q=.48 |  |
| Apo A-I: |  |  |  |  |  |  |  |
| HDL-1 | -0.04 (0.02), p=.07, q=.12 | -0.006 (0.02), p=.78, q=.96 | -0.05 (0.03), p=.05, q=.08 | -0.03 (0.03), p=.35, q=.65 | -0.07 (0.03), p=.007, q=.01 | -0.02 (0.02), p=.39, q=.84 |  |
| HDL-2 | -0.02 (0.02), p=.38, q=.46 | 0.01 (0.02), p=.51, q=.88 | -0.03 (0.03), p=.24, q=.31 | -0.01 (0.03), p=.72, q=.86 | -0.05 (0.03), p=.05, q=.07 | -0.005 (0.02), p=.86, q=.97 |  |
| HDL-3 | -0.04 (0.02), p=.12, q=.18 | -0.01 (0.02), p=.66, q=.96 | -0.04 (0.03), p=.10, q=.15 | -0.03 (0.03), p=.42, q=.68 | -0.07 (0.03), p=.02, q=.02 | 0.002 (0.03), p=.95, q=.99 |  |
| HDL-4 | -0.05 (0.02), p=.02, q=.05 | -0.006 (0.02), p=.77, q=.96 | -0.06 (0.02), p=.01, q=.03 | -0.06 (0.03), p=.05, q=.45 | -0.07 (0.02), p=.004, q=.007 | 0.008 (0.02), p=.74, q=.92 |  |
| HDL | -0.07 (0.02), p=.005, q=.02 | -0.006 (0.02), p=.79, q=.96 | -0.08 (0.03), p=.002, q=.009 | -0.06 (0.03), p=.06, q=.46 | -0.11 (0.03), p<.0001, q=.0001 | 0.00002 (0.02), p=.999, q=.999 |  |
| Total | -0.06 (0.02), p=.008, q=.02 | -0.003 (0.02), p=.89, q=.96 | -0.08 (0.03), p=.003, q=.01 | -0.06 (0.03), p=.06, q=.46 | -0.11 (0.03), p<.0001, q=.0002 | 0.002 (0.02), p=.93, q=.99 |  |
| Apo A-II: |  |  |  |  |  |  |  |
| HDL-1 | -0.02 (0.02), p=.48, q=.55 | -0.02 (0.02), p=.37, q=.84 | -0.01 (0.03), p=.57, q=.65 | 0.007 (0.03), p=.82, q=.94 | -0.03 (0.03), p=.23, q=.28 | -0.02 (0.03), p=.48, q=.84 |  |
| HDL-2 | -0.006 (0.02), p=.82, q=.84 | -0.01 (0.02), p=.63, q=.96 | -0.003 (0.03), p=.90, q=.91 | 0.02 (0.03), p=.63, q=.81 | -0.02 (0.03), p=.55, q=.59 | -0.02 (0.03), p=.47, q=.84 |  |
| HDL-3 | 0.01 (0.02), p=.59, q=.66 | 0.009 (0.02), p=.67, q=.96 | 0.01 (0.03), p=.62, q=.69 | 0.02 (0.03), p=.59, q=.79 | 0.008 (0.03), p=.76, q=.77 | 0.003 (0.02), p=.89, q=.97 |  |
| HDL-4 | -0.02 (0.02), p=.42, q=.50 | 0.005 (0.02), p=.82, q=.96 | -0.02 (0.02), p=.33, q=.39 | -0.04 (0.03), p=.20, q=.59 | -0.02 (0.02), p=.51, q=.56 | 0.01 (0.02), p=.56, q=.85 |  |
| HDL | -0.01 (0.02), p=.59, q=.66 | 0.02 (0.02), p=.39, q=.84 | -0.02 (0.03), p=.40, q=.46 | -0.02 (0.03), p=.42, q=.68 | -0.02 (0.03), p=.37, q=.42 | 0.009 (0.02), p=.72, q=.92 |  |
| Total | -0.02 (0.02), p=.40, q=.48 | 0.01 (0.02), p=.60, q=.94 | -0.03 (0.03), p=.27, q=.33 | -0.03 (0.03), p=.35, q=.65 | -0.03 (0.03), p=.23, q=.28 | 0.005 (0.02), p=.83, q=.97 |  |
| Apo B: |  |  |  |  |  |  |  |
| VLDL | 0.07 (0.02), p=.001, q=.006 | 0.03 (0.02), p=.13, q=.49 | 0.08 (0.02), p=.001, q=.006 | 0.03 (0.03), p=.33, q=.65 | 0.13 (0.02), p<.0001, q<.0001 | 0.04 (0.02), p=.09, q=.65 |  |
| IDL | 0.07 (0.02), p=.002, q=.0099 | 0.04 (0.02), p=.03, q=.25 | 0.07 (0.02), p=.004, q=.01 | 0.05 (0.03), p=.11, q=.49 | 0.09 (0.02), p=.0002, q=.0006 | 0.01 (0.02), p=.53, q=.84 |  |
| LDL-1 | 0.02 (0.02), p=.35, q=.44 | 0.02 (0.02), p=.24, q=.69 | 0.02 (0.03), p=.47, q=.54 | 0.02 (0.03), p=.58, q=.79 | 0.009 (0.03), p=.73, q=.75 | 0.03 (0.02), p=.20, q=.78 |  |
| LDL-2 | -0.03 (0.02), p=.23, q=.31 | 0.005 (0.02), p=.81, q=.96 | -0.04 (0.02), p=.16, q=.22 | -0.02 (0.03), p=.49, q=.73 | -0.06 (0.03), p=.02, q=.02 | 0.02 (0.02), p=.44, q=.84 |  |
| LDL-3 | -0.03 (0.02), p=.14, q=.20 | 0.006 (0.02), p=.75, q=.96 | -0.04 (0.02), p=.08, q=.13 | -0.02 (0.03), p=.42, q=.68 | -0.07 (0.02), p=.007, q=.01 | 0.003 (0.02), p=.90, q=.97 |  |
| LDL-4 | -0.01 (0.02), p=.58, q=.66 | -0.008 (0.02), p=.69, q=.96 | -0.01 (0.02), p=.60, q=.68 | 0.003 (0.03), p=.92, q=.99 | -0.02 (0.02), p=.34, q=.39 | -0.02 (0.02), p=.49, q=.84 |  |
| LDL-5 | 0.02 (0.02), p=.43, q=.51 | -0.01 (0.02), p=.52, q=.88 | 0.03 (0.02), p=.28, q=.34 | 0.01 (0.03), p=.70, q=.86 | 0.05 (0.02), p=.04, q=.06 | -0.02 (0.02), p=.44, q=.84 |  |
| LDL-6 | 0.03 (0.02), p=.12, q=.18 | 0.001 (0.02), p=.95, q=.97 | 0.04 (0.02), p=.08, q=.13 | 0.01 (0.03), p=.70, q=.86 | 0.07 (0.02), p=.004, q=.008 | 0.02 (0.02), p=.47, q=.84 |  |
| LDL | 0.008 (0.02), p=.71, q=.75 | -0.0002 (0.02), p=.99, q=.99 | 0.01 (0.02), p=.67, q=.74 | 0.0002 (0.03), p=.99, q=.999 | 0.02 (0.02), p=.48, q=.53 | 0.01 (0.02), p=.65, q=.88 |  |
| Total | 0.04 (0.02), p=.10, q=.16 | 0.02 (0.02), p=.44, q=.86 | 0.04 (0.02), p=.10, q=.15 | 0.01 (0.03), p=.67, q=.84 | 0.06 (0.02), p=.01, q=.02 | 0.03 (0.02), p=.27, q=.78 |  |

*Stabilization muscles include paraspinal, oblique, and rectus abdominus

Yellow shading indicates significant associations after accounting for multiple comparisons (FDR<1%).

Associations were adjusted for age, gender, race, alternate healthy eating index, moderate/vigorous physical activity, sedentary behavior, and lipid-lowering medication use.

**Supplementary Table 2.** Adjusted standardized regression estimates of the associations between 105 lipoprotein subfractions and **intermuscular adipose tissue (IMAT) area** of the total abdomen and locomotion (psoas), stabilization (paraspinal, oblique, and rectus abdominus), and individual muscle groups.

| **Lipoprotein subfractions:** | **Abdominal IMAT area** | | | | | |
| --- | --- | --- | --- | --- | --- | --- |
|  | **Total abdominal** | **Locomotion (psoas)** | **Stabilization*** | **Paraspinal** | **Oblique** | **Rectus abdominis** |
| Total  Cholesterol: |  |  |  |  |  |  |
| VLDL-1 | 0.09 (0.03), p=.002, q=.009 | 0.05 (0.03), p=.11, q=.22 | 0.09 (0.03), p=.002, q=.009 | 0.04 (0.03), p=.14, q=.48 | 0.12 (0.03), p=.0003, q=.001 | 0.11 (0.03), p=.001, q=.004 |
| VLDL-2 | 0.07 (0.03), p=.02, q=.04 | 0.02 (0.03), p=.47, q=.59 | 0.08 (0.03), p=.01, q=.03 | 0.03 (0.03), p=.31, q=.54 | 0.09 (0.03), p=.003, q=.01 | 0.11 (0.03), p=.001, q=.004 |
| VLDL-3 | 0.10 (0.03), p=.002, q=.008 | 0.06 (0.03), p=.06, q=.15 | 0.1 (0.03), p=.002, q=.009 | 0.04 (0.03), p=.22, q=.48 | 0.12 (0.03), p=.0002, q=.0009 | 0.12 (0.03), p=.0002, q=.0009 |
| VLDL-4 | 0.09 (0.03), p=.003, q=.01 | 0.07 (0.03), p=.04, q=.12 | 0.09 (0.03), p=.004, q=.01 | 0.03 (0.03), p=.30, q=.53 | 0.12 (0.03), p=.0001, q=.0007 | 0.11 (0.03), p=.0007, q=.003 |
| VLDL-5 | 0.08 (0.03), p=.007, q=.02 | 0.10 (0.03), p=.002, q=.05 | 0.07 (0.03), p=.02, q=.04 | 0.03 (0.03), p=.23, q=.48 | 0.09 (0.03), p=.006, q=.01 | 0.09 (0.03), p=.008, q=.02 |
| VLDL-6 | -0.08 (0.03), p=.01, q=.03 | -0.06 (0.03), p=.10, q=.20 | -0.07 (0.03), p=.01, q=.04 | -0.04 (0.03), p=.20, q=.48 | -0.09 (0.03), p=.005, q=.01 | -0.08 (0.03), p=.02, q=.04 |
| VLDL | 0.12 (0.03), p<.0001, q=.002 | 0.09 (0.03), p=.01, q=.07 | 0.12 (0.03), p=.0001, q=.003 | 0.05 (0.03), p=.09, q=.48 | 0.15 (0.03), p<.0001, q<.0001 | 0.15 (0.03), p<.0001, q<.0001 |
| IDL | 0.07 (0.03), p=.02, q=.04 | 0.03 (0.03), p=.34, q=.48 | 0.07 (0.03), p=.02, q=.04 | 0.04 (0.03), p=.18, q=.48 | 0.08 (0.03), p=.009, q=.02 | 0.08 (0.03), p=.01, q=.03 |
| LDL-1 | -0.002 (0.03), p=.96, q=.97 | -0.03 (0.03), p=.44, q=.59 | 0.002 (0.03), p=.95, q=.96 | 0.01 (0.03), p=.73, q=.90 | -0.01 (0.03), p=.76, q=.82 | -0.0001 (0.03), p=.997, q=.997 |
| LDL-2 | -0.04 (0.03), p=.20, q=.28 | -0.05 (0.03), p=.11, q=.22 | -0.04 (0.03), p=.26, q=.35 | -0.009 (0.03), p=.77, q=.92 | -0.05 (0.03), p=.17, q=.22 | -0.06 (0.03), p=.06, q=.09 |
| LDL-3 | -0.04 (0.03), p=.15, q=.23 | -0.07 (0.03), p=.03, q=.12 | -0.04 (0.03), p=.23, q=.33 | -0.006 (0.03), p=.84, q=.95 | -0.05 (0.03), p=.11, q=.16 | -0.07 (0.03), p=.04, q=.07 |
| LDL-4 | -0.009 (0.03), p=.77, q=.85 | -0.02 (0.03), p=.48, q=.59 | -0.006 (0.03), p=.84, q=.90 | 0.005 (0.03), p=.87, q=.95 | -0.007 (0.03), p=.82, q=.85 | -0.03 (0.03), p=.30, q=.35 |
| LDL-5 | 0.04 (0.03), p=.22, q=.29 | 0.07 (0.03), p=.04, q=.12 | 0.03 (0.03), p=.31, q=.40 | -0.004 (0.03), p=.89, q=.95 | 0.06 (0.03), p=.08, q=.12 | 0.05 (0.03), p=.10, q=.14 |
| LDL-6 | 0.04 (0.03), p=.17, q=.24 | 0.07 (0.03), p=.03, q=.12 | 0.03 (0.03), p=.26, q=.35 | -0.01 (0.03), p=.71, q=.90 | 0.07 (0.03), p=.02, q=.05 | 0.06 (0.03), p=.07, q=.10 |
| LDL | -0.005 (0.03), p=.88, q=.92 | -0.005 (0.03), p=.89, q=.93 | -0.004 (0.03), p=.89, q=.92 | -0.01 (0.03), p=.73, q=.90 | 0.008 (0.03), p=.80, q=.84 | -0.01 (0.03), p=.77, q=.83 |
| HDL-1 | -0.09 (0.03), p=.007, q=.02 | 0.009 (0.03), p=.80, q=.85 | -0.1 (0.03), p=.003, q=.01 | -0.06 (0.03), p=.06, q=.48 | -0.10 (0.03), p=.003, q=.01 | -0.10 (0.03), p=.003, q=.009 |
| HDL-2 | -0.09 (0.03), p=.01, q=.03 | -0.04 (0.04), p=.25, q=.40 | -0.09 (0.03), p=.01, q=.03 | -0.05 (0.03), p=.13, q=.48 | -0.09 (0.04), p=.01, q=.02 | -0.10 (0.04), p=.004, q=.009 |
| HDL-3 | -0.12 (0.03), p=.0005, q=.004 | -0.09 (0.04), p=.01, q=.08 | -0.11 (0.03), p=.0008, q=.005 | -0.06 (0.03), p=.05, q=.48 | -0.12 (0.03), p=.0005, q=.002 | -0.13 (0.04), p=.0002, q=.001 |
| HDL-4 | -0.05 (0.03), p=.10, q=.15 | -0.001 (0.03), p=.97, q=.98 | -0.05 (0.03), p=.07, q=.12 | -0.04 (0.03), p=.16, q=.48 | -0.04 (0.03), p=.17, q=.22 | -0.06 (0.03), p=.05, q=.09 |
| HDL | -0.11 (0.03), p=.001, q=.006 | -0.03 (0.04), p=.41, q=.56 | -0.11 (0.03), p=.0007, q=.005 | -0.07 (0.03), p=.03, q=.48 | -0.11 (0.03), p=.001, q=.005 | -0.13 (0.03), p=.0003, q=.001 |
| Total | 0.02 (0.03), p=.60, q=.70 | 0.007 (0.03), p=.83, q=.88 | 0.02 (0.03), p=.59, q=.68 | -0.003 (0.03), p=.91, q=.97 | 0.04 (0.03), p=.27, q=.34 | 0.02 (0.03), p=.54, q=.60 |
| Unesterified  Cholesterol: |  |  |  |  |  |  |
| VLDL-1 | 0.12 (0.03), p=.0002, q=.002 | 0.10 (0.03), p=.004, q=.05 | 0.11 (0.03), p=.0004, q=.003 | 0.05 (0.03), p=.11, q=.48 | 0.13 (0.03), p<.0001, q=.0004 | 0.15 (0.03), p<.0001, q<.0001 |
| VLDL-2 | 0.11 (0.03), p=.0007, q=.004 | 0.06 (0.03), p=.08, q=.17 | 0.11 (0.03), p=.0006, q=.005 | 0.05 (0.03), p=.11, q=.48 | 0.13 (0.03), p<.0001, q=.0005 | 0.13 (0.03), p<.0001, q=.0005 |
| VLDL-3 | 0.10 (0.03), p=.001, q=.005 | 0.07 (0.03), p=.04, q=.12 | 0.1 (0.03), p=.001, q=.006 | 0.03 (0.03), p=.24, q=.49 | 0.13 (0.03), p<.0001, q=.0004 | 0.14 (0.03), p<.0001, q=.0003 |
| VLDL-4 | 0.08 (0.03), p=.01, q=.03 | 0.05 (0.03), p=.17, q=.30 | 0.08 (0.03), p=.01, q=.03 | 0.03 (0.03), p=.23, q=.48 | 0.09 (0.03), p=.003, q=.01 | 0.10 (0.03), p=.003, q=.009 |
| VLDL-5 | 0.08 (0.03), p=.01, q=.03 | 0.08 (0.03), p=.01, q=.07 | 0.07 (0.03), p=.02, q=.04 | 0.03 (0.03), p=.38, q=.62 | 0.10 (0.03), p=.002, q=.008 | 0.09 (0.03), p=.004, q=.01 |
| VLDL-6 | 0.04 (0.03), p=.17, q=.24 | 0.06 (0.03), p=.05, q=.14 | 0.04 (0.03), p=.24, q=.34 | 0.01 (0.03), p=.67, q=.90 | 0.06 (0.03), p=.05, q=.09 | 0.01 (0.03), p=.70, q=.77 |
| VLDL | 0.13 (0.03), p<.0001, q=.002 | 0.10 (0.03), p=.004, q=.05 | 0.12 (0.03), p<.0001, q=.002 | 0.05 (0.03), p=.08, q=.48 | 0.15 (0.03), p<.0001, q<.0001 | 0.15 (0.03), p<.0001, q<.0001 |
| IDL | 0.08 (0.03), p=.01, q=.03 | 0.04 (0.03), p=.28, q=.43 | 0.08 (0.03), p=.01, q=.03 | 0.04 (0.03), p=.17, q=.48 | 0.09 (0.03), p=.005, q=.01 | 0.08 (0.03), p=.01, q=.03 |
| LDL-1 | -0.009 (0.03), p=.77, q=.85 | -0.02 (0.03), p=.61, q=.72 | -0.007 (0.03), p=.81, q=.89 | 0.007 (0.03), p=.82, q=.94 | -0.02 (0.03), p=.45, q=.53 | -0.006 (0.03), p=.86, q=.90 |
| LDL-2 | -0.06 (0.03), p=.06, q=.11 | -0.07 (0.03), p=.04, q=.12 | -0.05 (0.03), p=.09, q=.15 | -0.02 (0.03), p=.59, q=.86 | -0.07 (0.03), p=.04, q=.06 | -0.08 (0.03), p=.02, q=.03 |
| LDL-3 | -0.07 (0.03), p=.03, q=.06 | -0.09 (0.03), p=.005, q=.05 | -0.06 (0.03), p=.06, q=.11 | -0.01 (0.03), p=.64, q=.89 | -0.08 (0.03), p=.01, q=.02 | -0.09 (0.03), p=.006, q=.01 |
| LDL-4 | -0.03 (0.03), p=.37, q=.46 | -0.04 (0.03), p=.27, q=.43 | -0.02 (0.03), p=.43, q=.52 | 0.0001 (0.03), p=.997, q=.997 | -0.03 (0.03), p=.29, q=.35 | -0.06 (0.03), p=.08, q=.12 |
| LDL-5 | 0.02 (0.03), p=.60, q=.70 | 0.05 (0.03), p=.12, q=.24 | 0.01 (0.03), p=.75, q=.84 | -0.01 (0.03), p=.69, q=.90 | 0.03 (0.03), p=.36, q=.43 | 0.03 (0.03), p=.43, q=.49 |
| LDL-6 | 0.02 (0.03), p=.49, q=.58 | 0.05 (0.03), p=.14, q=.27 | 0.02 (0.03), p=.60, q=.69 | -0.02 (0.03), p=.53, q=.80 | 0.05 (0.03), p=.12, q=.18 | 0.03 (0.03), p=.30, q=.35 |
|  |  |  |  |  |  |  |
| Unesterified  Cholesterol cont.: |  |  |  |  |  |  |
| LDL | -0.03 (0.03), p=.30, q=.36 | -0.04 (0.03), p=.22, q=.36 | -0.03 (0.03), p=.35, q=.43 | -0.01 (0.03), p=.68, q=.90 | -0.03 (0.03), p=.28, q=.34 | -0.04 (0.03), p=.24, q=.29 |
| HDL-1 | -0.13 (0.03), p<.0001, q=.002 | -0.07 (0.03), p=.04, q=.12 | -0.12 (0.03), p<.0001, q=.002 | -0.06 (0.03), p=.04, q=.48 | -0.15 (0.03), p<.0001, q=.0001 | -0.14 (0.03), p<.0001, q=.0001 |
| HDL-2 | -0.12 (0.03), p=.0001, q=.002 | -0.12 (0.03), p=.0006, q=.03 | -0.12 (0.03), p=.0003, q=.003 | -0.05 (0.03), p=.07, q=.48 | -0.14 (0.03), p<.0001, q=.0002 | -0.14 (0.03), p<.0001, q=.0003 |
| HDL-3 | -0.10 (0.03), p=.0008, q=.005 | -0.12 (0.03), p=.0002, q=.02 | -0.09 (0.03), p=.002, q=.009 | -0.04 (0.03), p=.15, q=.48 | -0.11 (0.03), p=.0006, q=.002 | -0.12 (0.03), p=.0002, q=.001 |
| HDL-4 | -0.08 (0.03), p=.007, q=.02 | -0.08 (0.03), p=.02, q=.10 | -0.08 (0.03), p=.01, q=.03 | -0.04 (0.03), p=.17, q=.48 | -0.09 (0.03), p=.006, q=.01 | -0.10 (0.03), p=.003, q=.009 |
| HDL | -0.12 (0.03), p=.0002, q=.002 | -0.08 (0.03), p=.02, q=.08 | -0.11 (0.03), p=.0003, q=.003 | -0.05 (0.03), p=.10, q=.48 | -0.14 (0.03), p<.0001, q=.0002 | -0.14 (0.03), p<.0001, q=.0003 |
| Total | 0.005 (0.03), p=.87, q=.91 | -0.02 (0.03), p=.46, q=.59 | 0.009 (0.03), p=.77, q=.86 | 0.01 (0.03), p=.72, q=.90 | 0.007 (0.03), p=.83, q=.86 | -0.001 (0.03), p=.97, q=.98 |
| Phospholipids: |  |  |  |  |  |  |
| VLDL-1 | 0.11 (0.03), p=.0003, q=.002 | 0.10 (0.03), p=.004, q=.05 | 0.11 (0.03), p=.0005, q=.004 | 0.03 (0.03), p=.23, q=.48 | 0.14 (0.03), p<.0001, q=.0002 | 0.15 (0.03), p<.0001, q<.0001 |
| VLDL-2 | 0.11 (0.03), p=.0007, q=.004 | 0.07 (0.03), p=.03, q=.12 | 0.1 (0.03), p=.0009, q=.005 | 0.04 (0.03), p=.19, q=.48 | 0.13 (0.03), p<.0001, q=.0004 | 0.14 (0.03), p<.0001, q=.0002 |
| VLDL-3 | 0.10 (0.03), p=.0009, q=.005 | 0.07 (0.03), p=.03, q=.12 | 0.1 (0.03), p=.001, q=.006 | 0.04 (0.03), p=.21, q=.48 | 0.13 (0.03), p<.0001, q=.0004 | 0.14 (0.03), p<.0001, q=.0003 |
| VLDL-4 | 0.12 (0.03), p=.0001, q=.002 | 0.09 (0.03), p=.005, q=.05 | 0.11 (0.03), p=.0002, q=.003 | 0.04 (0.03), p=.13, q=.48 | 0.15 (0.03), p<.0001, q<.0001 | 0.14 (0.03), p<.0001, q=.0001 |
| VLDL-5 | 0.07 (0.03), p=.02, q=.04 | 0.07 (0.03), p=.03, q=.12 | 0.07 (0.03), p=.03, q=.06 | 0.03 (0.03), p=.26, q=.50 | 0.08 (0.03), p=.01, q=.02 | 0.08 (0.03), p=.02, q=.03 |
| VLDL-6 | 0.09 (0.03), p=.002, q=.009 | 0.06 (0.03), p=.06, q=.15 | 0.09 (0.03), p=.002, q=.01 | 0.04 (0.03), p=.19, q=.48 | 0.12 (0.03), p=.0002, q=.0009 | 0.11 (0.03), p=.001, q=.004 |
| VLDL | 0.12 (0.03), p<.0001, q=.002 | 0.10 (0.03), p=.002, q=.05 | 0.12 (0.03), p=.0002, q=.003 | 0.04 (0.03), p=.16, q=.48 | 0.15 (0.03), p<.0001, q<.0001 | 0.15 (0.03), p<.0001, q<.0001 |
| IDL | 0.06 (0.03), p=.05, q=.10 | 0.007 (0.03), p=.84, q=.88 | 0.07 (0.03), p=.03, q=.07 | 0.03 (0.03), p=.29, q=.53 | 0.08 (0.03), p=.01, q=.03 | 0.07 (0.03), p=.03, q=.06 |
| LDL-1 | 0.0008 (0.03), p=.98, q=.99 | -0.03 (0.03), p=.44, q=.59 | 0.005 (0.03), p=.88, q=.92 | 0.01 (0.03), p=.67, q=.90 | -0.009 (0.03), p=.78, q=.83 | 0.005 (0.03), p=.88, q=.91 |
| LDL-2 | -0.04 (0.03), p=.18, q=.25 | -0.06 (0.03), p=.07, q=.16 | -0.04 (0.03), p=.24, q=.34 | -0.008 (0.03), p=.78, q=.93 | -0.05 (0.03), p=.14, q=.19 | -0.06 (0.03), p=.06, q=.09 |
| LDL-3 | -0.05 (0.03), p=.13, q=.19 | -0.08 (0.03), p=.02, q=.10 | -0.04 (0.03), p=.20, q=.29 | -0.006 (0.03), p=.82, q=.94 | -0.06 (0.03), p=.08, q=.13 | -0.07 (0.03), p=.04, q=.07 |
| LDL-4 | -0.01 (0.03), p=.71, q=.79 | -0.03 (0.03), p=.39, q=.54 | -0.008 (0.03), p=.79, q=.87 | 0.004 (0.03), p=.88, q=.95 | -0.01 (0.03), p=.71, q=.78 | -0.03 (0.03), p=.30, q=.35 |
| LDL-5 | 0.03 (0.03), p=.25, q=.33 | 0.06 (0.03), p=.06, q=.15 | 0.03 (0.03), p=.35, q=.43 | -0.005 (0.03), p=.86, q=.95 | 0.05 (0.03), p=.09, q=.14 | 0.05 (0.03), p=.11, q=.15 |
| LDL-6 | 0.04 (0.03), p=.23, q=.31 | 0.07 (0.03), p=.04, q=.12 | 0.03 (0.03), p=.33, q=.41 | -0.01 (0.03), p=.61, q=.87 | 0.07 (0.03), p=.03, q=.06 | 0.06 (0.03), p=.08, q=.12 |
| LDL | -0.008 (0.03), p=.80, q=.86 | -0.01 (0.03), p=.68, q=.77 | -0.006 (0.03), p=.84, q=.90 | -0.01 (0.03), p=.72, q=.90 | 0.004 (0.03), p=.91, q=.92 | -0.009 (0.03), p=.79, q=.85 |
| HDL-1 | -0.07 (0.03), p=.05, q=.10 | 0.02 (0.04), p=.60, q=.71 | -0.07 (0.03), p=.03, q=.06 | -0.04 (0.03), p=.15, q=.48 | -0.08 (0.03), p=.02, q=.05 | -0.07 (0.04), p=.04, q=.06 |
| HDL-2 | -0.10 (0.03), p=.005, q=.02 | -0.05 (0.04), p=.17, q=.30 | -0.1 (0.03), p=.005, q=.02 | -0.05 (0.03), p=.10, q=.48 | -0.10 (0.04), p=.004, q=.01 | -0.11 (0.04), p=.003, q=.009 |
| HDL-3 | -0.09 (0.03), p=.009, q=.02 | -0.07 (0.04), p=.04, q=.12 | -0.09 (0.03), p=.01, q=.03 | -0.05 (0.03), p=.15, q=.48 | -0.10 (0.04), p=.008, q=.02 | -0.10 (0.04), p=.006, q=.02 |
| HDL-4 | -0.06 (0.03), p=.07, q=.11 | -0.02 (0.03), p=.57, q=.69 | -0.06 (0.03), p=.06, q=.11 | -0.04 (0.03), p=.15, q=.48 | -0.05 (0.03), p=.12, q=.17 | -0.06 (0.03), p=.06, q=.09 |
| HDL | -0.11 (0.03), p=.002, q=.007 | -0.05 (0.04), p=.20, q=.33 | -0.11 (0.03), p=.001, q=.006 | -0.06 (0.03), p=.06, q=.48 | -0.12 (0.04), p=.0008, q=.003 | -0.12 (0.04), p=.001, q=.004 |
| Triglycerides: |  |  |  |  |  |  |
| VLDL-1 | 0.11 (0.03), p=.0006, q=.004 | 0.10 (0.03), p=.002, q=.05 | 0.1 (0.03), p=.001, q=.006 | 0.03 (0.03), p=.37, q=.61 | 0.14 (0.03), p<.0001, q=.0002 | 0.15 (0.03), p<.0001, q<.0001 |
| VLDL-2 | 0.10 (0.03), p=.0009, q=.005 | 0.06 (0.03), p=.06, q=.15 | 0.1 (0.03), p=.001, q=.006 | 0.04 (0.03), p=.15, q=.48 | 0.13 (0.03), p=.0001, q=.0006 | 0.13 (0.03), p<.0001, q=.0004 |
| VLDL-3 | 0.10 (0.03), p=.002, q=.008 | 0.06 (0.03), p=.08, q=.17 | 0.1 (0.03), p=.002, q=.009 | 0.04 (0.03), p=.20, q=.48 | 0.12 (0.03), p=.0002, q=.0009 | 0.12 (0.03), p=.0002, q=.001 |
| VLDL-4 | 0.11 (0.03), p=.0002, q=.002 | 0.09 (0.03), p=.006, q=.05 | 0.11 (0.03), p=.0003, q=.003 | 0.04 (0.03), p=.15, q=.48 | 0.14 (0.03), p<.0001, q=.0001 | 0.14 (0.03), p<.0001, q=.0001 |
| VLDL-5 | 0.08 (0.03), p=.007, q=.02 | 0.09 (0.03), p=.01, q=.07 | 0.08 (0.03), p=.01, q=.03 | 0.04 (0.03), p=.19, q=.48 | 0.10 (0.03), p=.003, q=.01 | 0.08 (0.03), p=.01, q=.02 |
| VLDL-6 | 0.002 (0.03), p=.94, q=.97 | -0.01 (0.03), p=.75, q=.81 | 0.004 (0.03), p=.90, q=.92 | -0.009 (0.03), p=.76, q=.92 | 0.02 (0.03), p=.58, q=.64 | 0.01 (0.03), p=.75, q=.82 |
| VLDL | 0.12 (0.03), p=.0001, q=.002 | 0.09 (0.03), p=.005, q=.05 | 0.12 (0.03), p=.0002, q=.003 | 0.04 (0.03), p=.15, q=.48 | 0.15 (0.03), p<.0001, q<.0001 | 0.15 (0.03), p<.0001, q<.0001 |
| IDL | 0.08 (0.03), p=.02, q=.04 | 0.05 (0.03), p=.14, q=.27 | 0.07 (0.03), p=.02, q=.04 | 0.03 (0.03), p=.30, q=.53 | 0.09 (0.03), p=.007, q=.02 | 0.10 (0.03), p=.002, q=.006 |
| LDL-1 | 0.06 (0.03), p=.06, q=.11 | 0.03 (0.03), p=.45, q=.59 | 0.06 (0.03), p=.05, q=.10 | 0.02 (0.03), p=.42, q=.66 | 0.07 (0.03), p=.03, q=.05 | 0.09 (0.03), p=.008, q=.02 |
| LDL-2 | -0.008 (0.03), p=.79, q=.86 | -0.07 (0.03), p=.04, q=.12 | 0.001 (0.03), p=.97, q=.97 | 0.02 (0.03), p=.53, q=.80 | -0.02 (0.03), p=.46, q=.53 | 0.001 (0.03), p=.97, q=.98 |
| LDL-3 | 0.02 (0.03), p=.63, q=.72 | -0.04 (0.03), p=.19, q=.33 | 0.02 (0.03), p=.47, q=.56 | 0.03 (0.03), p=.29, q=.53 | -0.002 (0.03), p=.94, q=.94 | 0.03 (0.03), p=.43, q=.49 |
| LDL-4 | 0.02 (0.03), p=.42, q=.51 | -0.03 (0.03), p=.34, q=.48 | 0.03 (0.03), p=.31, q=.4 | 0.02 (0.03), p=.41, q=.66 | 0.02 (0.03), p=.56, q=.63 | 0.05 (0.03), p=.13, q=.17 |
| LDL-5 | 0.06 (0.03), p=.06, q=.11 | 0.04 (0.03), p=.23, q=.38 | 0.06 (0.03), p=.06, q=.11 | 0.01 (0.03), p=.61, q=.87 | 0.07 (0.03), p=.03, q=.05 | 0.10 (0.03), p=.003, q=.009 |
| LDL-6 | 0.06 (0.03), p=.06, q=.11 | 0.04 (0.03), p=.17, q=.3 | 0.05 (0.03), p=.07, q=.12 | -0.0005 (0.03), p=.99, q=.997 | 0.09 (0.03), p=.004, q=.01 | 0.09 (0.03), p=.004, q=.009 |
| LDL | 0.06 (0.03), p=.04, q=.09 | 0.01 (0.03), p=.74, q=.81 | 0.07 (0.03), p=.03, q=.07 | 0.03 (0.03), p=.23, q=.48 | 0.07 (0.03), p=.03, q=.05 | 0.08 (0.03), p=.01, q=.03 |
| Triglycerides cont.: |  |  |  |  |  |  |
| HDL-1 | 0.03 (0.03), p=.29, q=.36 | 0.03 (0.03), p=.34, q=.49 | 0.03 (0.03), p=.31, q=.40 | 0.03 (0.03), p=.39, q=.62 | 0.02 (0.03), p=.48, q=.55 | 0.04 (0.03), p=.30, q=.35 |
| HDL-2 | 0.05 (0.03), p=.15, q=.22 | 0.01 (0.03), p=.75, q=.81 | 0.05 (0.03), p=.13, q=.20 | 0.03 (0.03), p=.26, q=.50 | 0.04 (0.03), p=.20, q=.26 | 0.05 (0.03), p=.11, q=.15 |
| HDL-3 | 0.05 (0.03), p=.10, q=.15 | 0.002 (0.03), p=.95, q=.97 | 0.06 (0.03), p=.07, q=.12 | 0.04 (0.03), p=.23, q=.48 | 0.06 (0.03), p=.09, q=.14 | 0.06 (0.03), p=.06, q=.09 |
| HDL-4 | 0.08 (0.03), p=.009, q=.02 | 0.06 (0.03), p=.08, q=.17 | 0.08 (0.03), p=.01, q=.03 | 0.04 (0.03), p=.22, q=.48 | 0.09 (0.03), p=.004, q=.01 | 0.10 (0.03), p=.002, q=.006 |
| HDL | 0.07 (0.03), p=.03, q=.07 | 0.05 (0.03), p=.15, q=.28 | 0.07 (0.03), p=.04, q=.08 | 0.04 (0.03), p=.17, q=.48 | 0.06 (0.03), p=.05, q=.09 | 0.08 (0.03), p=.02, q=.04 |
| Total | 0.12 (0.03), p=.0001, q=.002 | 0.09 (0.03), p=.009, q=.07 | 0.12 (0.03), p=.0002, q=.003 | 0.05 (0.03), p=.11, q=.48 | 0.15 (0.03), p<.0001, q=.0001 | 0.14 (0.03), p<.0001, q=.0002 |
| Apo A-I: |  |  |  |  |  |  |
| HDL-1 | -0.04 (0.03), p=.25, q=.33 | 0.01 (0.03), p=.68, q=.77 | -0.04 (0.03), p=.19, q=.29 | -0.007 (0.03), p=.83, q=.94 | -0.06 (0.03), p=.06, q=.10 | -0.06 (0.03), p=.06, q=.09 |
| HDL-2 | -0.06 (0.03), p=.07, q=.11 | -0.07 (0.04), p=.05, q=.13 | -0.06 (0.03), p=.09, q=.15 | -0.02 (0.03), p=.59, q=.86 | -0.08 (0.03), p=.03, q=.05 | -0.08 (0.04), p=.03, q=.06 |
| HDL-3 | -0.08 (0.03), p=.02, q=.04 | -0.07 (0.04), p=.05, q=.14 | -0.08 (0.03), p=.02, q=.05 | -0.04 (0.03), p=.22, q=.48 | -0.09 (0.04), p=.02, q=.03 | -0.09 (0.04), p=.01, q=.02 |
| HDL-4 | -0.03 (0.03), p=.29, q=.36 | 0.01 (0.03), p=.75, q=.81 | -0.04 (0.03), p=.23, q=.33 | -0.03 (0.03), p=.29, q=.53 | -0.02 (0.03), p=.45, q=.53 | -0.04 (0.03), p=.18, q=.22 |
| HDL | -0.09 (0.03), p=.006, q=.02 | -0.02 (0.04), p=.52, q=.64 | -0.1 (0.03), p=.004, q=.01 | -0.06 (0.03), p=.06, q=.48 | -0.10 (0.03), p=.006, q=.01 | -0.11 (0.03), p=.002, q=.005 |
| Total | -0.08 (0.03), p=.01, q=.03 | -0.01 (0.04), p=.69, q=.78 | -0.09 (0.03), p=.007, q=.02 | -0.06 (0.03), p=.07, q=.48 | -0.09 (0.03), p=.01, q=.03 | -0.10 (0.03), p=.003, q=.009 |
| Apo A-II: |  |  |  |  |  |  |
| HDL-1 | -0.06 (0.03), p=.08, q=.14 | -0.0007 (0.04), p=.98, q=.98 | -0.06 (0.03), p=.06, q=.11 | -0.04 (0.03), p=.21, q=.48 | -0.07 (0.03), p=.05, q=.09 | -0.06 (0.04), p=.11, q=.15 |
| HDL-2 | -0.06 (0.03), p=.09, q=.14 | -0.04 (0.04), p=.31, q=.46 | -0.06 (0.03), p=.09, q=.15 | -0.04 (0.03), p=.16, q=.48 | -0.05 (0.04), p=.17, q=.23 | -0.05 (0.04), p=.16, q=.20 |
| HDL-3 | -0.05 (0.03), p=.11, q=.17 | -0.07 (0.03), p=.04, q=.13 | -0.05 (0.03), p=.16, q=.24 | -0.03 (0.03), p=.35, q=.59 | -0.05 (0.03), p=.18, q=.23 | -0.05 (0.03), p=.17, q=.22 |
| HDL-4 | -0.01 (0.03), p=.67, q=.76 | 0.02 (0.03), p=.46, q=.59 | -0.02 (0.03), p=.57, q=.66 | -0.03 (0.03), p=.29, q=.53 | 0.005 (0.03), p=.88, q=.89 | -0.007 (0.03), p=.84, q=.88 |
| HDL | -0.06 (0.03), p=.06, q=.11 | -0.04 (0.03), p=.29, q=.43 | -0.06 (0.03), p=.06, q=.11 | -0.05 (0.03), p=.09, q=.48 | -0.04 (0.03), p=.20, q=.25 | -0.06 (0.03), p=.09, q=.13 |
| Total | -0.06 (0.03), p=.06, q=.11 | -0.03 (0.03), p=.39, q=.55 | -0.06 (0.03), p=.05, q=.10 | -0.05 (0.03), p=.10, q=.48 | -0.05 (0.03), p=.16, q=.22 | -0.06 (0.03), p=.06, q=.09 |
| Apo B: |  |  |  |  |  |  |
| VLDL | 0.13 (0.03), p<.0001, q=.002 | 0.09 (0.03), p=.005, q=.05 | 0.13 (0.03), p<.0001, q=.002 | 0.05 (0.03), p=.06, q=.48 | 0.15 (0.03), p<.0001, q<.0001 | 0.16 (0.03), p<.0001, q<.0001 |
| IDL | 0.08 (0.03), p=.007, q=.02 | 0.02 (0.03), p=.60, q=.71 | 0.09 (0.03), p=.004, q=.01 | 0.05 (0.03), p=.07, q=.48 | 0.09 (0.03), p=.005, q=.01 | 0.09 (0.03), p=.004, q=.01 |
| LDL-1 | -0.00002 (0.03), p=.9995, q=.9995 | -0.04 (0.03), p=.27, q=.43 | 0.005 (0.03), p=.86, q=.92 | 0.01 (0.03), p=.63, q=.89 | -0.01 (0.03), p=.75, q=.82 | 0.007 (0.03), p=.83, q=.88 |
| LDL-2 | -0.03 (0.03), p=.29, q=.36 | -0.06 (0.03), p=.08, q=.17 | -0.03 (0.03), p=.39, q=.47 | -0.0004 (0.03), p=.99, q=.997 | -0.04 (0.03), p=.20, q=.26 | -0.05 (0.03), p=.12, q=.16 |
| LDL-3 | -0.04 (0.03), p=.20, q=.28 | -0.08 (0.03), p=.02, q=.10 | -0.03 (0.03), p=.31, q=.40 | -0.002 (0.03), p=.96, q=.99 | -0.05 (0.03), p=.13, q=.19 | -0.06 (0.03), p=.09, q=.12 |
| LDL-4 | -0.007 (0.03), p=.81, q=.86 | -0.04 (0.03), p=.28, q=.43 | -0.003 (0.03), p=.93, q=.95 | 0.008 (0.03), p=.78, q=.93 | -0.009 (0.03), p=.78, q=.83 | -0.02 (0.03), p=.51, q=.58 |
| LDL-5 | 0.04 (0.03), p=.17, q=.24 | 0.06 (0.03), p=.05, q=.14 | 0.04 (0.03), p=.23, q=.33 | -0.002 (0.03), p=.95, q=.99 | 0.06 (0.03), p=.05, q=.09 | 0.06 (0.03), p=.05, q=.08 |
| LDL-6 | 0.05 (0.03), p=.09, q=.15 | 0.07 (0.03), p=.03, q=.12 | 0.04 (0.03), p=.14, q=.22 | -0.005 (0.03), p=.86, q=.95 | 0.08 (0.03), p=.009, q=.02 | 0.07 (0.03), p=.03, q=.05 |
| LDL | 0.02 (0.03), p=.52, q=.61 | 0.002 (0.03), p=.94, q=.97 | 0.02 (0.03), p=.49, q=.58 | 0.0005 (0.03), p=.99, q=.997 | 0.04 (0.03), p=.26, q=.33 | 0.03 (0.03), p=.32, q=.38 |
| Total | 0.06 (0.03), p=.06, q=.11 | 0.02 (0.03), p=.47, q=.59 | 0.06 (0.03), p=.06, q=.11 | 0.02 (0.03), p=.52, q=.80 | 0.08 (0.03), p=.01, q=.03 | 0.07 (0.03), p=.02, q=.04 |

*Stabilization muscles include paraspinal, oblique, and rectus abdominus

Yellow shading indicates significant associations after accounting for multiple comparisons (FDR<1%).

Associations were adjusted for age, gender, race, alternate healthy eating index, moderate/vigorous physical activity, sedentary behavior, and lipid-lowering medication use.

**Supplementary Table 3.** Adjusted standardized regression estimates of the associations between 105 lipoprotein subfractions and **intermuscular adipose tissue (IMAT) density** of the total abdomen and locomotion (psoas), stabilization (paraspinal, oblique, and rectus abdominus), and individual muscle groups.

| **Lipoprotein** **subfractions:** | **Abdominal IMAT density** | | | | | |
| --- | --- | --- | --- | --- | --- | --- |
|  | **Total** | **Locomotion (psoas)** | **Stabilization**** | **Paraspinal** | **Oblique** | **Rectus abdominis** |
| Total  Cholesterol: |  |  |  |  |  |  |
| VLDL-1 | -0.14 (0.03), p<.0001, q<.0001 | -0.11 (0.03), p=.0007, q=.003 | -0.12 (0.03), p=.0001, q=.0005 | 0.01 (0.03), p=.69, q=.75 | -0.12 (0.03), p=.0003, q=.001 | -0.06 (0.03), p=.10, q=.23 |
| VLDL-2 | -0.13 (0.03), p=.0001, q=.0005 | -0.09 (0.03), p=.005, q=.02 | -0.12 (0.03), p=.0003, q=.001 | 0.05 (0.03), p=.11, q=.19 | -0.12 (0.03), p=.0004, q=.002 | -0.06 (0.03), p=.06, q=.17 |
| VLDL-3 | -0.14 (0.03), p<.0001, q<.0001 | -0.10 (0.03), p=.002, q=.008 | -0.14 (0.03), p<.0001, q=.0002 | 0.06 (0.03), p=.07, q=.13 | -0.14 (0.03), p<.0001, q=.0001 | -0.09 (0.03), p=.005, q=.03 |
| VLDL-4 | -0.13 (0.03), p<.0001, q=.0001 | -0.10 (0.03), p=.003, q=.01 | -0.13 (0.03), p<.0001, q=.0004 | 0.001 (0.03), p=.97, q=.98 | -0.12 (0.03), p=.0001, q=.0006 | -0.09 (0.03), p=.004, q=.03 |
| VLDL-5 | -0.11 (0.03), p=.0004, q=.001 | -0.11 (0.03), p=.0007, q=.004 | -0.09 (0.03), p=.003, q=.01 | -0.10 (0.03), p=.001, q=.02 | -0.05 (0.03), p=.09, q=.20 | -0.09 (0.03), p=.01, q=.04 |
| VLDL-6 | 0.11 (0.03), p=.0005, q=.002 | 0.11 (0.03), p=.0006, q=.003 | 0.09 (0.03), p=.005, q=.01 | -0.06 (0.03), p=.07, q=.13 | 0.09 (0.03), p=.006, q=.02 | 0.06 (0.03), p=.08, q=.22 |
| VLDL | -0.17 (0.03), p<.0001, q<.0001 | -0.13 (0.03), p=.0001, q=.0009 | -0.15 (0.03), p<.0001, q<.0001 | 0.07 (0.04), p=.05, q=.12 | -0.15 (0.03), p<.0001, q<.0001 | -0.11 (0.03), p=.0006, q=.03 |
| IDL | -0.12 (0.03), p=.0002, q=.0008 | -0.08 (0.03), p=.01, q=.03 | -0.11 (0.03), p=.0005, q=.002 | 0.06 (0.03), p=.08, q=.15 | -0.10 (0.03), p=.003, q=.01 | -0.07 (0.03), p=.03, q=.09 |
| LDL-1 | 0.008 (0.03), p=.80, q=.83 | 0.02 (0.03), p=.48, q=.57 | 0.00004 (0.03), p=.999, q=.999 | -0.09 (0.03), p=.006, q=.05 | -0.007 (0.03), p=.84, q=.93 | -0.002 (0.03), p=.96, q=.98 |
| LDL-2 | 0.07 (0.03), p=.03, q=.05 | 0.07 (0.03), p=.04, q=.08 | 0.06 (0.03), p=.06, q=.11 | -0.07 (0.03), p=.02, q=.07 | 0.03 (0.03), p=.39, q=.56 | 0.05 (0.03), p=.14, q=.30 |
| LDL-3 | 0.08 (0.03), p=.02, q=.04 | 0.08 (0.03), p=.01, q=.03 | 0.06 (0.03), p=.07, q=.12 | -0.09 (0.03), p=.008, q=.05 | 0.03 (0.03), p=.43, q=.60 | 0.06 (0.03), p=.09, q=.22 |
| LDL-4 | 0.04 (0.03), p=.22, q=.30 | 0.05 (0.03), p=.14, q=.22 | 0.03 (0.03), p=.38, q=.50 | -0.08 (0.03), p=.02, q=.06 | 0.004 (0.03), p=.91, q=.96 | 0.05 (0.03), p=.16, q=.33 |
| LDL-5 | -0.05 (0.03), p=.12, q=.17 | -0.06 (0.03), p=.07, q=.13 | -0.04 (0.03), p=.25, q=.35 | 0.07 (0.04), p=.06, q=.13 | -0.02 (0.03), p=.47, q=.62 | -0.03 (0.03), p=.39, q=.58 |
| LDL-6 | -0.07 (0.03), p=.02, q=.05 | -0.09 (0.03), p=.008, q=.02 | -0.05 (0.03), p=.10, q=.17 | 0.02 (0.03), p=.52, q=.60 | -0.04 (0.03), p=.22, q=.37 | -0.04 (0.03), p=.23, q=.41 |
| LDL | 0.02 (0.03), p=.51, q=.58 | 0.02 (0.03), p=.58, q=.64 | 0.02 (0.03), p=.56, q=.65 | -0.04 (0.03), p=.25, q=.36 | -0.003 (0.03), p=.93, q=.96 | 0.02 (0.03), p=.52, q=.65 |
| HDL-1 | 0.11 (0.03), p=.001, q=.003 | 0.03 (0.03), p=.32, q=.43 | 0.12 (0.03), p=.0003, q=.001 | -0.08 (0.03), p=.009, q=.05 | 0.12 (0.03), p=.0005, q=.002 | 0.08 (0.03), p=.02, q=.07 |
| HDL-2 | 0.07 (0.04), p=.04, q=.07 | 0.02 (0.04), p=.50, q=.58 | 0.08 (0.04), p=.02, q=.05 | -0.10 (0.03), p=.002, q=.03 | 0.07 (0.04), p=.04, q=.10 | 0.04 (0.04), p=.23, q=.41 |
| HDL-3 | 0.06 (0.04), p=.08, q=.13 | 0.03 (0.04), p=.32, q=.44 | 0.06 (0.04), p=.08, q=.15 | 0.03 (0.03), p=.32, q=.43 | 0.04 (0.04), p=.27, q=.44 | 0.02 (0.04), p=.57, q=.71 |
| HDL-4 | -0.01 (0.03), p=.68, q=.73 | -0.03 (0.03), p=.40, q=.52 | -0.004 (0.03), p=.89, q=.92 | 0.05 (0.03), p=.12, q=.20 | -0.03 (0.03), p=.31, q=.48 | -0.009 (0.03), p=.79, q=.88 |
| HDL | 0.07 (0.03), p=.04, q=.06 | 0.02 (0.03), p=.64, q=.69 | 0.08 (0.03), p=.02, q=.04 | -0.03 (0.03), p=.34, q=.46 | 0.06 (0.03), p=.08, q=.18 | 0.05 (0.04), p=.17, q=.33 |
| Total | -0.04 (0.03), p=.18, q=.24 | -0.04 (0.03), p=.26, q=.38 | -0.04 (0.03), p=.23, q=.34 | 0.05 (0.04), p=.19, q=.28 | -0.05 (0.03), p=.10, q=.21 | -0.01 (0.03), p=.70, q=.82 |
| Unesterified Cholesterol: |  |  |  |  |  |  |
| VLDL-1 | -0.17 (0.03), p<.0001, q<.0001 | -0.14 (0.03), p<.0001, q=.0002 | -0.15 (0.03), p<.0001, q<.0001 | 0.07 (0.03), p=.04, q=.09 | -0.13 (0.03), p<.0001, q=.0005 | -0.12 (0.03), p=.0004, q=.03 |
| VLDL-2 | -0.15 (0.03), p<.0001, q<.0001 | -0.11 (0.03), p=.0005, q=.003 | -0.14 (0.03), p<.0001, q=.0002 | 0.07 (0.03), p=.03, q=.07 | -0.15 (0.03), p<.0001, q<.0001 | -0.09 (0.03), p=.007, q=.04 |
| VLDL-3 | -0.15 (0.03), p<.0001, q<.0001 | -0.11 (0.03), p=.001, q=.004 | -0.14 (0.03), p<.0001, q=.0002 | 0.02 (0.03), p=.61, q=.69 | -0.15 (0.03), p<.0001, q<.0001 | -0.09 (0.03), p=.007, q=.04 |
| VLDL-4 | -0.12 (0.03), p=.0003, q=.001 | -0.09 (0.03), p=.008, q=.02 | -0.11 (0.03), p=.0007, q=.003 | -0.02 (0.03), p=.47, q=.57 | -0.10 (0.03), p=.003, q=.01 | -0.07 (0.03), p=.03, q=.09 |
| VLDL-5 | -0.12 (0.03), p=.0002, q=.0009 | -0.10 (0.03), p=.003, q=.009 | -0.11 (0.03), p=.001, q=.004 | -0.07 (0.03), p=.02, q=.07 | -0.08 (0.03), p=.02, q=.06 | -0.09 (0.03), p=.006, q=.03 |
| VLDL | -0.19 (0.03), p<.0001, q<.0001 | -0.16 (0.03), p<.0001, q=.0001 | -0.17 (0.03), p<.0001, q<.0001 | 0.09 (0.03), p=.004, q=.05 | -0.16 (0.03), p<.0001, q<.0001 | -0.11 (0.03), p=.002, q=.03 |
| IDL | -0.11 (0.03), p=.0005, q=.002 | -0.07 (0.03), p=.04, q=.09 | -0.11 (0.03), p=.0005, q=.002 | -0.03 (0.03), p=.30, q=.41 | -0.10 (0.03), p=.002, q=.006 | -0.08 (0.03), p=.02, q=.08 |
| LDL-1 | 0.02 (0.03), p=.58, q=.65 | 0.03 (0.03), p=.45, q=.55 | 0.01 (0.03), p=.73, q=.80 | -0.07 (0.03), p=.03, q=.08 | 0.008 (0.03), p=.81, q=.93 | 0.0002 (0.03), p=.996, q=.996 |
| LDL-2 | 0.09 (0.03), p=.009, q=.02 | 0.08 (0.03), p=.02, q=.05 | 0.07 (0.03), p=.02, q=.05 | -0.07 (0.03), p=.04, q=.09 | 0.04 (0.03), p=.26, q=.43 | 0.06 (0.03), p=.09, q=.23 |
| LDL-3 | 0.10 (0.03), p=.003, q=.007 | 0.10 (0.03), p=.003, q=.01 | 0.08 (0.03), p=.01, q=.04 | -0.09 (0.03), p=.006, q=.05 | 0.05 (0.03), p=.16, q=.30 | 0.07 (0.03), p=.04, q=.13 |
| LDL-4 | 0.06 (0.03), p=.06, q=.10 | 0.06 (0.03), p=.07, q=.13 | 0.05 (0.03), p=.12, q=.19 | 0.07 (0.03), p=.03, q=.08 | 0.03 (0.03), p=.43, q=.60 | 0.06 (0.03), p=.06, q=.17 |
| LDL-5 | -0.02 (0.03), p=.56, q=.63 | -0.03 (0.03), p=.30, q=.41 | -0.009 (0.03), p=.79, q=.84 | 0.04 (0.03), p=.25, q=.36 | 0.003 (0.03), p=.93, q=.96 | -0.006 (0.03), p=.85, q=.94 |
| LDL-6 | -0.06 (0.03), p=.08, q=.13 | -0.07 (0.03), p=.03, q=.07 | -0.04 (0.03), p=.23, q=.34 | -0.08 (0.03), p=.02, q=.06 | -0.03 (0.03), p=.35, q=.51 | -0.02 (0.03), p=.49, q=.64 |
|  |  |  |  |  |  |  |
| Unesterified Cholesterol  cont.: |  |  |  |  |  |  |
| LDL | 0.06 (0.03), p=.08, q=.13 | 0.06 (0.03), p=.09, q=.15 | 0.05 (0.03), p=.16, q=.24 | -0.02 (0.03), p=.63, q=.70 | 0.02 (0.03), p=.48, q=.62 | 0.05 (0.03), p=.17, q=.33 |
| HDL-1 | 0.15 (0.03), p<.0001, q<.0001 | 0.10 (0.03), p=.002, q=.009 | 0.14 (0.03), p<.0001, q=.0001 | -0.11 (0.03), p=.0005, q=.02 | 0.12 (0.03), p=.0003, q=.001 | 0.11 (0.03), p=.002, q=.03 |
| HDL-2 | 0.12 (0.03), p=.0004, q=.001 | 0.10 (0.03), p=.002, q=.009 | 0.1 (0.03), p=.002, q=.006 | -0.02 (0.03), p=.49, q=.59 | 0.08 (0.03), p=.02, q=.06 | 0.07 (0.03), p=.04, q=.13 |
| HDL-3 | 0.07 (0.03), p=.04, q=.06 | 0.08 (0.03), p=.01, q=.03 | 0.05 (0.03), p=.13, q=.20 | 0.03 (0.03), p=.37, q=.49 | 0.02 (0.03), p=.53, q=.65 | 0.04 (0.03), p=.21, q=.39 |
| HDL-4 | 0.03 (0.03), p=.43, q=.53 | 0.03 (0.03), p=.44, q=.55 | 0.02 (0.03), p=.52, q=.63 | 0.07 (0.03), p=.03, q=.08 | -0.01 (0.03), p=.69, q=.82 | 0.03 (0.03), p=.41, q=.58 |
| HDL | 0.11 (0.03), p=.002, q=.004 | 0.08 (0.03), p=.01, q=.03 | 0.1 (0.03), p=.004, q=.01 | 0.03 (0.03), p=.39, q=.49 | 0.07 (0.03), p=.03, q=.09 | 0.08 (0.03), p=.03, q=.09 |
| Total | -0.02 (0.03), p=.60, q=.67 | 0.0002 (0.03), p=.99, q=.99 | -0.02 (0.03), p=.51, q=.62 | 0.09 (0.03), p=.01, q=.05 | -0.04 (0.03), p=.28, q=.44 | 0.01 (0.03), p=.71, q=.82 |
| Phospholipids: |  |  |  |  |  |  |
| VLDL-1 | -0.16 (0.03), p<.0001, q<.0001 | -0.14 (0.03), p<.0001, q=.0004 | -0.14 (0.03), p<.0001, q=.0001 | 0.02 (0.03), p=.60, q=.68 | -0.14 (0.03), p<.0001, q=.0002 | -0.11 (0.03), p=.001, q=.03 |
| VLDL-2 | -0.16 (0.03), p<.0001, q<.0001 | -0.13 (0.03), p<.0001, q=.0009 | -0.14 (0.03), p<.0001, q=.0001 | 0.05 (0.03), p=.11, q=.19 | -0.14 (0.03), p<.0001, q=.0002 | -0.09 (0.03), p=.006, q=.03 |
| VLDL-3 | -0.15 (0.03), p<.0001, q<.0001 | -0.12 (0.03), p=.0004, q=.002 | -0.14 (0.03), p<.0001, q=.0001 | 0.06 (0.03), p=.07, q=.13 | -0.15 (0.03), p<.0001, q<.0001 | -0.09 (0.03), p=.006, q=.03 |
| VLDL-4 | -0.16 (0.03), p<.0001, q<.0001 | -0.13 (0.03), p<.0001, q=.0009 | -0.14 (0.03), p<.0001, q=.0001 | 0.008 (0.03), p=.82, q=.88 | -0.13 (0.03), p<.0001, q=.0003 | -0.10 (0.03), p=.002, q=.03 |
| VLDL-5 | -0.11 (0.03), p=.0008, q=.002 | -0.09 (0.03), p=.004, q=.01 | -0.09 (0.03), p=.003, q=.01 | 0.06 (0.03), p=.06, q=.13 | -0.07 (0.03), p=.03, q=.07 | -0.09 (0.03), p=.008, q=.04 |
| VLDL-6 | -0.15 (0.03), p<.0001, q<.0001 | -0.13 (0.03), p=.0001, q=.0009 | -0.13 (0.03), p<.0001, q=.0002 | -0.08 (0.03), p=.02, q=.06 | -0.13 (0.03), p<.0001, q=.0003 | -0.06 (0.03), p=.07, q=.21 |
| VLDL | -0.17 (0.03), p<.0001, q<.0001 | -0.15 (0.03), p<.0001, q=.0001 | -0.15 (0.03), p<.0001, q<.0001 | 0.07 (0.04), p=.06, q=.12 | -0.14 (0.03), p<.0001, q=.0002 | -0.10 (0.03), p=.002, q=.03 |
| IDL | -0.10 (0.03), p=.002, q=.004 | -0.06 (0.03), p=.06, q=.12 | -0.1 (0.03), p=.002, q=.006 | 0.08 (0.03), p=.02, q=.06 | -0.10 (0.03), p=.004, q=.01 | -0.05 (0.03), p=.14, q=.3 |
| LDL-1 | 0.006 (0.03), p=.87, q=.88 | 0.02 (0.03), p=.49, q=.57 | -0.003 (0.03), p=.92, q=.95 | -0.07 (0.03), p=.03, q=.08 | -0.006 (0.03), p=.86, q=.94 | -0.004 (0.03), p=.92, q=.97 |
| LDL-2 | 0.08 (0.03), p=.02, q=.04 | 0.08 (0.03), p=.02, q=.05 | 0.06 (0.03), p=.06, q=.11 | -0.07 (0.03), p=.03, q=.08 | 0.03 (0.03), p=.35, q=.51 | 0.05 (0.03), p=.13, q=.29 |
| LDL-3 | 0.08 (0.03), p=.01, q=.03 | 0.09 (0.03), p=.007, q=.02 | 0.06 (0.03), p=.06, q=.11 | -0.09 (0.03), p=.008, q=.05 | 0.03 (0.03), p=.36, q=.53 | 0.06 (0.03), p=.08, q=.21 |
| LDL-4 | 0.04 (0.03), p=.20, q=.27 | 0.05 (0.03), p=.10, q=.17 | 0.03 (0.03), p=.39, q=.50 | -0.11 (0.03), p=.001, q=.02 | 0.007 (0.03), p=.82, q=.93 | 0.05 (0.03), p=.17, q=.33 |
| LDL-5 | -0.05 (0.03), p=.15, q=.21 | -0.05 (0.03), p=.11, q=.18 | -0.03 (0.03), p=.28, q=.38 | 0.08 (0.03), p=.02, q=.07 | -0.02 (0.03), p=.51, q=.63 | -0.03 (0.03), p=.42, q=.59 |
| LDL-6 | -0.07 (0.03), p=.04, q=.07 | -0.08 (0.03), p=.01, q=.03 | -0.05 (0.03), p=.15, q=.22 | 0.02 (0.03), p=.45, q=.57 | -0.03 (0.03), p=.29, q=.46 | -0.04 (0.03), p=.28, q=.46 |
| LDL | 0.02 (0.03), p=.45, q=.53 | 0.03 (0.03), p=.44, q=.55 | 0.02 (0.03), p=.54, q=.64 | -0.04 (0.03), p=.21, q=.31 | 0.003 (0.03), p=.94, q=.96 | 0.02 (0.03), p=.51, q=.65 |
| HDL-1 | 0.08 (0.03), p=.02, q=.04 | 0.01 (0.04), p=.71, q=.76 | 0.1 (0.03), p=.004, q=.01 | -0.08 (0.03), p=.008, q=.05 | 0.10 (0.04), p=.004, q=.01 | 0.06 (0.04), p=.07, q=.21 |
| HDL-2 | 0.08 (0.04), p=.04, q=.07 | 0.04 (0.04), p=.28, q=.40 | 0.08 (0.04), p=.03, q=.07 | -0.09 (0.03), p=.008, q=.05 | 0.07 (0.04), p=.05, q=.11 | 0.03 (0.04), p=.43, q=.59 |
| HDL-3 | 0.03 (0.04), p=.35, q=.44 | 0.02 (0.04), p=.56, q=.63 | 0.03 (0.04), p=.36, q=.48 | -0.02 (0.03), p=.51, q=.60 | 0.03 (0.04), p=.49, q=.62 | -0.002 (0.04), p=.96, q=.98 |
| HDL-4 | -0.02 (0.03), p=.45, q=.53 | -0.02 (0.03), p=.51, q=.58 | -0.02 (0.03), p=.51, q=.62 | -0.05 (0.03), p=.10, q=.18 | -0.04 (0.03), p=.18, q=.34 | -0.03 (0.03), p=.45, q=.60 |
| HDL | 0.07 (0.04), p=.07, q=.12 | 0.03 (0.04), p=.45, q=.55 | 0.07 (0.04), p=.05, q=.10 | -0.03 (0.03), p=.32, q=.43 | 0.06 (0.04), p=.11, q=.22 | 0.03 (0.04), p=.40, q=.58 |
| Triglycerides: |  |  |  |  |  |  |
| VLDL-1 | -0.17 (0.03), p<.0001, q<.0001 | -0.15 (0.03), p<.0001, q=.0001 | -0.15 (0.03), p<.0001, q<.0001 | -0.01 (0.03), p=.69, q=.75 | -0.14 (0.03), p<.0001, q=.0001 | -0.11 (0.03), p=.002, q=.03 |
| VLDL-2 | -0.16 (0.03), p<.0001, q<.0001 | -0.13 (0.03), p=.0001, q=.0009 | -0.14 (0.03), p<.0001, q=.0001 | -0.08 (0.03), p=.009, q=.05 | -0.15 (0.03), p<.0001, q<.0001 | -0.08 (0.03), p=.01, q=.06 |
| VLDL-3 | -0.15 (0.03), p<.0001, q<.0001 | -0.12 (0.03), p=.0002, q=.002 | -0.14 (0.03), p<.0001, q=.0002 | -0.11 (0.03), p=.001, q=.02 | -0.15 (0.03), p<.0001, q<.0001 | -0.08 (0.03), p=.02, q=.07 |
| VLDL-4 | -0.16 (0.03), p<.0001, q<.0001 | -0.14 (0.03), p<.0001, q=.0002 | -0.14 (0.03), p<.0001, q=.0001 | -0.05 (0.03), p=.10, q=.18 | -0.14 (0.03), p<.0001, q=.0002 | -0.09 (0.03), p=.005, q=.03 |
| VLDL-5 | -0.12 (0.03), p=.0004, q=.001 | -0.11 (0.03), p=.0009, q=.004 | -0.1 (0.03), p=.004, q=.01 | -0.006 (0.03), p=.86, q=.90 | -0.07 (0.03), p=.05, q=.11 | -0.08 (0.03), p=.03, q=.09 |
| VLDL-6 | -0.006 (0.03), p=.87, q=.88 | 0.004 (0.03), p=.91, q=.92 | -0.009 (0.03), p=.79, q=.84 | 0.02 (0.03), p=.47, q=.57 | -0.04 (0.03), p=.24, q=.40 | 0.02 (0.03), p=.47, q=.63 |
| VLDL | -0.18 (0.03), p<.0001, q<.0001 | -0.16 (0.03), p<.0001, q=.0001 | -0.16 (0.03), p<.0001, q<.0001 | 0.007 (0.03), p=.83, q=.89 | -0.16 (0.03), p<.0001, q<.0001 | -0.09 (0.03), p=.006, q=.03 |
| IDL | -0.15 (0.03), p<.0001, q<.0001 | -0.11 (0.03), p=.0006, q=.003 | -0.14 (0.03), p<.0001, q=.0001 | 0.05 (0.03), p=.15, q=.25 | -0.15 (0.03), p<.0001, q<.0001 | -0.10 (0.03), p=.004, q=.03 |
| LDL-1 | -0.08 (0.03), p=.02, q=.05 | -0.06 (0.03), p=.09, q=.16 | -0.07 (0.03), p=.04, q=.08 | 0.05 (0.04), p=.17, q=.26 | -0.04 (0.03), p=.21, q=.37 | -0.03 (0.03), p=.40, q=.58 |
| LDL-2 | 0.02 (0.03), p=.51, q=.58 | 0.06 (0.03), p=.07, q=.13 | 0.0003 (0.03), p=.99, q=.999 | 0.06 (0.03), p=.09, q=.18 | 0.003 (0.03), p=.92, q=.96 | -0.004 (0.03), p=.92, q=.97 |
| LDL-3 | 0.03 (0.03), p=.37, q=.47 | 0.06 (0.03), p=.07, q=.13 | 0.01 (0.03), p=.77, q=.84 | 0.03 (0.03), p=.41, q=.52 | 0.02 (0.03), p=.49, q=.62 | 0.02 (0.03), p=.49, q=.64 |
| LDL-4 | -0.02 (0.03), p=.62, q=.68 | 0.02 (0.03), p=.61, q=.66 | -0.03 (0.03), p=.39, q=.50 | 0.03 (0.03), p=.38, q=.49 | -0.007 (0.03), p=.83, q=.93 | -0.002 (0.03), p=.96, q=.98 |
| LDL-5 | -0.08 (0.03), p=.02, q=.04 | -0.06 (0.03), p=.05, q=.10 | -0.07 (0.03), p=.03, q=.07 | 0.003 (0.03), p=.91, q=.95 | -0.04 (0.03), p=.22, q=.37 | -0.05 (0.03), p=.16, q=.33 |
| LDL-6 | -0.09 (0.03), p=.005, q=.01 | -0.08 (0.03), p=.01, q=.03 | -0.08 (0.03), p=.02, q=.04 | 0.04 (0.04), p=.27, q=.38 | -0.07 (0.03), p=.04, q=.09 | -0.03 (0.03), p=.39, q=.58 |
| LDL | -0.07 (0.03), p=.02, q=.05 | -0.05 (0.03), p=.17, q=.26 | -0.07 (0.03), p=.02, q=.05 | 0.05 (0.03), p=.11, q=.19 | -0.06 (0.03), p=.09, q=.20 | -0.04 (0.03), p=.29, q=.46 |
| Triglycerides cont.: |  |  |  |  |  |  |
| HDL-1 | -0.03 (0.03), p=.31, q=.40 | -0.03 (0.03), p=.38, q=.50 | -0.03 (0.03), p=.38, q=.49 | 0.02 (0.03), p=.52, q=.60 | -0.01 (0.03), p=.70, q=.82 | -0.01 (0.03), p=.78, q=.88 |
| HDL-2 | -0.05 (0.03), p=.10, q=.15 | -0.03 (0.03), p=.36, q=.47 | -0.06 (0.03), p=.10, q=.17 | 0.05 (0.03), p=.16, q=.26 | -0.04 (0.03), p=.24, q=.40 | -0.03 (0.03), p=.41, q=.58 |
| HDL-3 | -0.09 (0.03), p=.01, q=.02 | -0.05 (0.03), p=.18, q=.26 | -0.09 (0.03), p=.008, q=.02 | 0.05 (0.03), p=.11, q=.19 | -0.07 (0.03), p=.03, q=.07 | -0.06 (0.03), p=.09, q=.23 |
| HDL-4 | -0.15 (0.03), p<.0001, q<.0001 | -0.11 (0.03), p=.0006, q=.003 | -0.14 (0.03), p<.0001, q=.0001 | 0.05 (0.03), p=.16, q=.25 | -0.13 (0.03), p<.0001, q=.0005 | -0.09 (0.03), p=.01, q=.04 |
| HDL | -0.09 (0.03), p=.006, q=.02 | -0.07 (0.03), p=.03, q=.07 | -0.08 (0.03), p=.01, q=.03 | -0.003 (0.03), p=.93, q=.95 | -0.06 (0.03), p=.06, q=.13 | -0.04 (0.03), p=.24, q=.41 |
| Total | -0.18 (0.03), p<.0001, q<.0001 | -0.15 (0.03), p<.0001, q=.0001 | -0.16 (0.03), p<.0001, q<.0001 | -0.04 (0.03), p=.23, q=.34 | -0.16 (0.03), p<.0001, q<.0001 | -0.09 (0.03), p=.007, q=.04 |
| Apo A-I: |  |  |  |  |  |  |
| HDL-1 | 0.05 (0.03), p=.13, q=.19 | 0.02 (0.03), p=.48, q=.57 | 0.05 (0.03), p=.12, q=.19 | -0.05 (0.03), p=.10, q=.18 | 0.06 (0.03), p=.10, q=.20 | 0.01 (0.04), p=.68, q=.81 |
| HDL-2 | 0.03 (0.03), p=.45, q=.53 | 0.04 (0.04), p=.30, q=.41 | 0.02 (0.04), p=.63, q=.72 | -0.04 (0.03), p=.21, q=.31 | 0.02 (0.04), p=.63, q=.76 | -0.003 (0.04), p=.94, q=.98 |
| HDL-3 | 0.01 (0.04), p=.73, q=.76 | 0.008 (0.04), p=.83, q=.86 | 0.01 (0.04), p=.73, q=.80 | -0.07 (0.03), p=.02, q=.07 | -0.005 (0.04), p=.88, q=.94 | -0.01 (0.04), p=.78, q=.88 |
| HDL-4 | -0.05 (0.03), p=.13, q=.18 | -0.05 (0.03), p=.13, q=.21 | -0.04 (0.03), p=.22, q=.32 | -0.07 (0.03), p=.03, q=.08 | -0.07 (0.03), p=.03, q=.08 | -0.03 (0.03), p=.44, q=.59 |
| HDL | 0.03 (0.03), p=.46, q=.55 | -0.009 (0.04), p=.80, q=.84 | 0.04 (0.04), p=.30, q=.40 | -0.07 (0.03), p=.04, q=.09 | 0.006 (0.04), p=.88, q=.94 | 0.02 (0.04), p=.60, q=.73 |
| Total | 0.02 (0.03), p=.65, q=.71 | -0.02 (0.04), p=.59, q=.65 | 0.03 (0.03), p=.41, q=.52 | -0.10 (0.03), p=.002, q=.03 | -0.001 (0.04), p=.97, q=.98 | 0.01 (0.04), p=.69, q=.81 |
| Apo A-II: |  |  |  |  |  |  |
| HDL-1 | 0.08 (0.04), p=.02, q=.05 | 0.02 (0.04), p=.48, q=.57 | 0.09 (0.04), p=.01, q=.03 | 0.0003 (0.03), p=.99, q=.99 | 0.09 (0.04), p=.008, q=.02 | 0.05 (0.04), p=.15, q=.33 |
| HDL-2 | 0.04 (0.04), p=.28, q=.37 | 0.02 (0.04), p=.60, q=.66 | 0.04 (0.04), p=.26, q=.36 | -0.08 (0.03), p=.01, q=.06 | 0.04 (0.04), p=.32, q=.49 | 0.004 (0.04), p=.92, q=.97 |
| HDL-3 | 0.003 (0.03), p=.94, q=.94 | 0.006 (0.03), p=.85, q=.88 | 0.0004 (0.03), p=.99, q=.999 | -0.003 (0.03), p=.94, q=.95 | -0.01 (0.03), p=.77, q=.90 | -0.02 (0.04), p=.63, q=.76 |
| HDL-4 | -0.07 (0.03), p=.03, q=.05 | -0.08 (0.03), p=.01, q=.03 | -0.05 (0.03), p=.10, q=.17 | -0.08 (0.03), p=.02, q=.06 | -0.08 (0.03), p=.02, q=.06 | -0.04 (0.03), p=.20, q=.37 |
| HDL | -0.03 (0.03), p=.34, q=.44 | -0.04 (0.03), p=.26, q=.38 | -0.02 (0.03), p=.49, q=.61 | -0.02 (0.03), p=.51, q=.60 | -0.05 (0.03), p=.11, q=.22 | -0.03 (0.03), p=.37, q=.57 |
| Total | -0.02 (0.03), p=.47, q=.55 | -0.04 (0.03), p=.26, q=.38 | -0.01 (0.03), p=.69, q=.77 | -0.05 (0.03), p=.12, q=.20 | -0.04 (0.03), p=.19, q=.35 | -0.02 (0.03), p=.58, q=.71 |
| Apo B: |  |  |  |  |  |  |
| VLDL | -0.18 (0.03), p<.0001, q<.0001 | -0.15 (0.03), p<.0001, q=.0001 | -0.16 (0.03), p<.0001, q<.0001 | -0.08 (0.03), p=.02, q=.06 | -0.15 (0.03), p<.0001, q<.0001 | -0.11 (0.03), p=.001, q=.03 |
| IDL | -0.12 (0.03), p=.0002, q=.0007 | -0.06 (0.03), p=.07, q=.13 | -0.13 (0.03), p<.0001, q=.0004 | -0.09 (0.03), p=.006, q=.05 | -0.11 (0.03), p=.001, q=.005 | -0.10 (0.03), p=.003, q=.03 |
| LDL-1 | 0.004 (0.03), p=.90, q=.91 | 0.03 (0.03), p=.43, q=.55 | -0.007 (0.03), p=.84, q=.88 | -0.08 (0.03), p=.01, q=.06 | -0.008 (0.03), p=.81, q=.93 | -0.004 (0.03), p=.90, q=.97 |
| LDL-2 | 0.07 (0.03), p=.04, q=.07 | 0.07 (0.03), p=.03, q=.07 | 0.05 (0.03), p=.11, q=.17 | -0.07 (0.03), p=.02, q=.07 | 0.02 (0.03), p=.46, q=.62 | 0.04 (0.03), p=.19, q=.36 |
| LDL-3 | 0.07 (0.03), p=.02, q=.05 | 0.08 (0.03), p=.009, q=.03 | 0.06 (0.03), p=.09, q=.15 | -0.08 (0.03), p=.01, q=.06 | 0.02 (0.03), p=.45, q=.61 | 0.06 (0.03), p=.10, q=.23 |
| LDL-4 | 0.03 (0.03), p=.30, q=.39 | 0.05 (0.03), p=.11, q=.18 | 0.02 (0.03), p=.56, q=.65 | -0.11 (0.03), p=.001, q=.02 | -0.0009 (0.03), p=.98, q=.98 | 0.04 (0.03), p=.24, q=.41 |
| LDL-5 | -0.06 (0.03), p=.07, q=.12 | -0.06 (0.03), p=.06, q=.12 | -0.04 (0.03), p=.17, q=.25 | -0.11 (0.03), p=.0004, q=.02 | -0.03 (0.03), p=.39, q=.56 | -0.04 (0.03), p=.28, q=.46 |
| LDL-6 | -0.08 (0.03), p=.008, q=.02 | -0.09 (0.03), p=.004, q=.01 | -0.07 (0.03), p=.04, q=.08 | 0.02 (0.03), p=.60, q=.68 | -0.05 (0.03), p=.13, q=.25 | -0.05 (0.03), p=.16, q=.33 |
| LDL | -0.01 (0.03), p=.69, q=.73 | -0.005 (0.03), p=.88, q=.90 | -0.01 (0.03), p=.66, q=.75 | -0.09 (0.03), p=.006, q=.05 | -0.02 (0.03), p=.59, q=.72 | -0.001 (0.03), p=.97, q=.98 |
| Total | -0.07 (0.03), p=.03, q=.06 | -0.05 (0.03), p=.14, q=.22 | -0.07 (0.03), p=.04, q=.08 | -0.09 (0.03), p=.007, q=.05 | -0.07 (0.03), p=.04, q=.10 | -0.03 (0.03), p=.37, q=.57 |

*Stabilization muscles include paraspinal, oblique, and rectus abdominus

Yellow shading indicates significant associations after accounting for multiple comparisons (FDR<1%).

Associations were adjusted for age, gender, race, alternate healthy eating index, moderate/vigorous physical activity, sedentary behavior, and lipid-lowering medication use.
